# *Arabidopsis thaliana RESISTANCE TO FUSARIUM OXYSPORUM 2* Implicates Tyrosine-Sulfated Peptide Signaling in Susceptibility and Resistance to Root Infection

**DOI:** 10.1371/journal.pgen.1003525

**Published:** 2013-05-23

**Authors:** Yunping Shen, Andrew C. Diener

**Affiliations:** Molecular, Cell and Developmental Biology, University of California Los Angeles, Los Angeles, California, United States of America; Virginia Tech, United States of America

## Abstract

In the plant *Arabidopsis thaliana*, multiple quantitative trait loci (QTLs), including *RFO2*, account for the strong resistance of accession Columbia-0 (Col-0) and relative susceptibility of Taynuilt-0 (Ty-0) to the vascular wilt fungus *Fusarium oxysporum* forma specialis *matthioli*. We find that *RFO2* corresponds to diversity in receptor-like protein (RLP) genes. In Col-0, there is a tandem pair of RLP genes: *RFO2*/At1g17250 confers resistance while *RLP2* does not. In Ty-0, the highly diverged *RFO2* locus has one RLP gene conferring weaker resistance. While the endogenous *RFO2* makes a modest contribution to resistance, transgenic *RFO2* provides strong pathogen-specific resistance. The extracellular leucine-rich repeats (eLRRs) in RFO2 and RLP2 are interchangeable for resistance and remarkably similar to eLRRs in the receptor-like kinase PSY1R, which perceives tyrosine-sulfated peptide PSY1. Reduced infection in *psy1r* and mutants of related phytosulfokine (PSK) receptor genes *PSKR1* and *PSKR2* shows that tyrosine-sulfated peptide signaling promotes susceptibility. The related eLRRs in RFO2 and PSY1R are not interchangeable; and expression of the RLP nPcR, in which eLRRs in RFO2 are replaced with eLRRs in PSY1R, results in constitutive resistance. Counterintuitively, PSY1 signaling suppresses *nPcR* because *psy1r nPcR* is lethal. The fact that PSK signaling does not similarly affect *nPcR* argues that PSY1 signaling directly downregulates the expression of *nPcR*. Our results support a speculative but intriguing model to explain *RFO2*'s role in resistance. We propose that *F. oxysporum* produces an effector that inhibits the normal negative feedback regulation of PSY1R, which stabilizes PSY1 signaling and induces susceptibility. However, RFO2, acting as a decoy receptor for PSY1R, is also stabilized by the effector and instead induces host immunity. Overall, the quantitative resistance of *RFO2* is reminiscent of the better-studied monogenic resistance traits.

**Authors Summary**

The fungus *Fusarium oxysporum* causes debilitating vascular infections in plants and is responsible for Fusarium wilt diseases in numerous crop species. To cope with microbial pathogens such as *F. oxysporum*, plants express variation in resistance genes, which typically facilitate recognition of infection by pathogens and instigate a defense response. Presently, receptor like-proteins (RLPs) are characterized as a minor class of resistance proteins with strong effect. Studying resistance to Fusarium wilt disease in the plant *Arabidopsis thaliana*, we discover that *RFO2*, a gene providing modest quantitative resistance, encodes an RLP. Extracellular leucine-rich repeats (eLRRs) of RLPs typically mediate the recognition of infection by pathogens. However, we find that the eLRRs of RFO2 do not specify resistance. The eLRRs of RFO2 and PSY1R, which is the putative receptor for an endogenous tyrosine-sulfated peptide growth regulator PSY1, share remarkable identity. Moreover, we find that PSY1 signaling promotes susceptibility to Fusarium wilt disease. From genetic analysis of a novel RLP gene that we created from both *RFO2* and *PSY1R*, we propose a model that explains the relationship between *RFO2* and *PSY1R*. In our model, *RFO2* induces resistance because *RFO2* mediates the recognition of *F. oxysporum*'s attempt to manipulate PSY1 signaling.

## Introduction

The fungus *Fusarium oxysporum* largely persists in soil as a saprophyte or in the roots of asymptomatic plants as an endophyte [1], [2]. It is the rarer pathogens of *F. oxysporum* that are capable of invading and colonizing the vascular system of host plants, and persistence of *F. oxysporum* in water-conducting xylem vessels is indicative of host susceptibility [1]–[4]. Numerous agricultural crops, notably tomato, cotton and banana, are susceptible to debilitating vascular infection by *F. oxysporum* and consequently develop wilt disease [2], [3], [5], [6].

Fusarium wilt diseases can be especially destructive to crop monocultures because pathogens are virulent in a narrow range of plant species [3], [7]. In recognition of this host specificity, pathogenic isolates are classified as having special forms, or formae speciales, which typically represent one to several phylogenetic lineages in the *F. oxysporum* species complex [7]. Pathogens of the same forma specialis infect similar host species, and a commercial host often names the forma specialis. For instance, *F. oxysporum* forma specialis *matthioli* (FOM) is isolated from garden stock (*Matthiola incana*) [8].

Fusarium wilt of *Arabidopsis thaliana* is an ideal pathosystem for mapping, identifying and characterizing the genes responsible for host resistance to vascular wilt fungi [9]. *A. thaliana* is the preeminent plant for molecular genetic and genomic studies and is susceptible to infection by FOM and two other crucifer-infecting formae speciales [9], [10]. In the field, *F. oxysporum* forma specialis *conglutinans* (FOC) and *F. oxysporum* forma specialis *raphani* (FOR) are recovered from diseased cabbage (*Brassica* species) and radish (*Raphanus sativus*), respectively [11]. The symptoms and progression of wilt disease in *A. thaliana* recapitulate the disease syndrome observed in native field hosts [8], [9], [12], [13]. Furthermore, this experimental pathosystem preserves host specificity because *A. thaliana* remains completely resistant to formae speciales isolated from non-crucifer hosts [9].

Innate resistance to Fusarium wilt as well as other infectious diseases often varies among plants of the same or interbreeding species [9], [14], [15]. Host resistance to the infecting pathogen when available in commercially acceptable varieties and crop rotation when feasible are preferable measures to control soil-borne diseases such as Fusarium wilt because chemical treatment of fields is usually uneconomical or has too negative cost to the environment [3], [14], [16]. However, genetic resistance may be poorly defined or unavailable in acceptable crop varieties.

The response of wild accessions of *A. thaliana* to infection by FOC, FOM and FOR ranges widely from complete resistance to ready susceptibility [9]. For example, accession Col-0 exhibits complete resistance to a dose of FOM that consistently kills accession Ty-0. On the other hand, Ty-0 exhibits more resistance than Col-0 when accessions are instead infected with FOC race 1. Thus, in large part, variation in resistance is specific to the infecting forma specialis. Most researchers using the *Fusarium*-*Arabidopsis* pathosystem infect the common laboratory accession Col-0 with FOC [17]–[19]. Because Col-0 exhibits considerable but partial resistance to FOC, it is possible to observe either enhanced resistance or increased susceptibility in *Arabidopsis* mutants using the same *F. oxysporum* pathogen.

To improve the resistance of cultivated varieties, plant breeders exploit the genes controlling natural variation in resistance, so-called resistance genes [20]. In crosses between resistant and susceptible varieties, qualitative resistance may be inherited as multiple quantitative trait loci (QTLs) conferring polygenic resistance or as a simple discontinuous Mendelian trait conferring monogenic resistance [21]. The best-studied resistance genes confer strong monogenic resistance to specific pathogens and typically but not always code for members of the nucleotide binding, leucine-rich repeat (NB-LRR) class of resistance proteins [22], [23]. There are few examples of genes providing polygenic resistance, so it remains unclear whether particular classes of genes with common function are commonly associated with quantitative disease resistance traits [24]–[26].

In *A. thaliana*, *RESISTANCE TO F. OXYSPORUM* (*RFO*) is a polygenic trait [9]. Six *RFO* QTLs are detected in the recombinant progeny of Col-0 and Ty-0 accessions and account for the strong resistance of Col-0 and susceptibility of Ty-0 to FOM. *RFO1*, which expresses the strongest resistance among *RFO* QTLs, is a member of the wall-associated kinase (WAK) family of receptor-like kinase (RLK) genes. The WAK family is one of several RLK gene families whose history, genome organization and expression suggest their involvement in response to pathogens [27]. *RFO1* contributes quantitatively to immunity as loss-of-function in *rfo1* enhances *F. oxysporum* infection in the root vascular cylinder [28]. Resistance conferred by *RFO2* and two other *RFO* QTLs appears epistatic to *RFO1* and is either enhanced or dependent on the presence of *RFO1* [9].

Here we show that the *RFO2* QTL corresponds to diversity in receptor-like protein (RLP) genes that have conspicuous sequence similarity to the PSY1 peptide receptor gene *PSY1R* [29]. We find that, while the native *RFO2* in Col-0 expresses modest quantitative resistance, transgenic *RFO2* expresses strong, nearly qualitative resistance and confers specific resistance to FOM and no resistance to FOC. In contrast, we find that the *RFO2*-related *PSY1R* and phytosulfokine (PSK) receptor genes *PSKR1* and *PSKR2* promote susceptibility to *F. oxysporum* infection [30]. From the phenotypes and genetic interactions of chimeric RLP and RLK transgenes, we characterize the resistance function of *RFO2* and propose a speculative model that connects the peptide signaling of *PSY1R* and pathogen-specific resistance of *RFO2*.

## Results

### Mapping and identification of *RFO2*

In previous genetic analysis, resistance to FOM was associated with six *RFO* loci in recombinant offspring of Col-0 and Ty-0 accessions of *A. thaliana* [9]. Plants that were heterozygous Col-0/Ty-0 at *RFO* loci, including *RFO2* on chromosome 1, exhibited more resistance than plants that were homozygous Ty-0/Ty-0.

**Figure 1:**
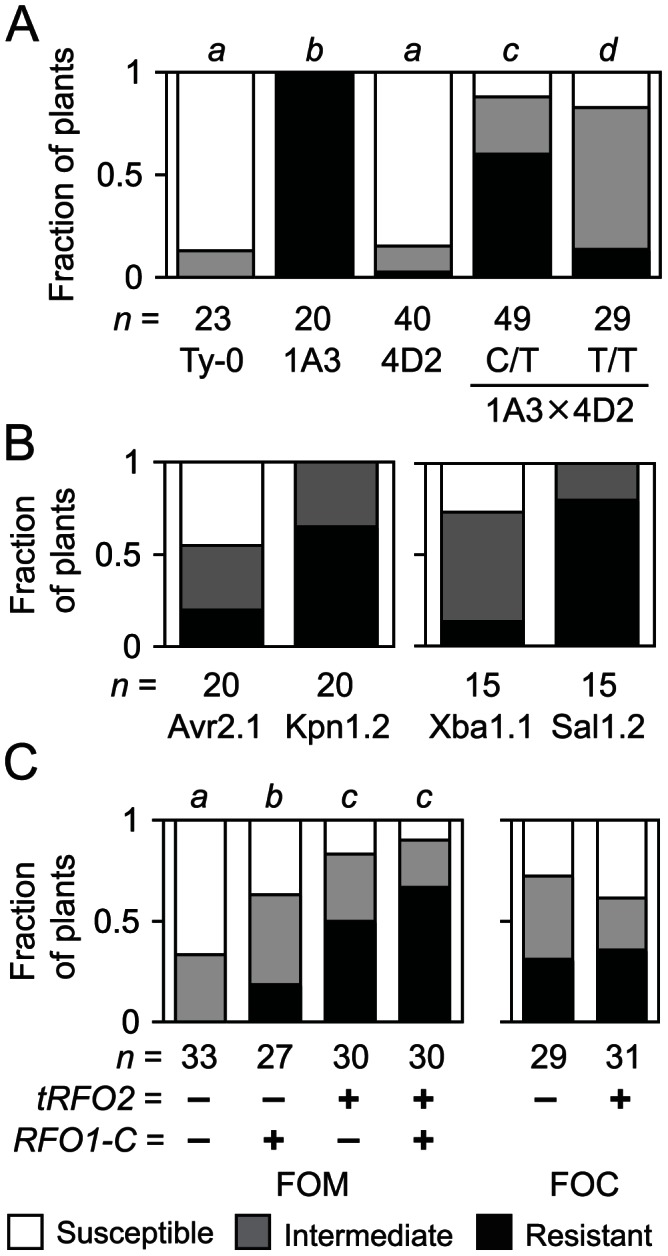
Resistance of endogenous and transgenic *RFO2*. (A) Fractions of *n* plants, either Ty-0, line 1A3, self progeny of 4D2 (*RFO2-C/T*), or progeny of cross 1A3×4D2, *RFO2-C/T* (C/T) or *RFO2-T/T* (T/T), were susceptible or resistant, or had intermediate resistance, according to HI scores at 18 dpi. The same italicized letter is for genotypes with similar median ranks, according to the Mann-Whitney (M-W) *U* test (two-tailed *p*>0.01) of ranks from HI scores at three time points in one week. (B) Fractions of *n* T_1_ 1A3 harboring Col-0 genomic clone Kpn1.2, Avr2.1, Xba1.1 or Sal1.2 were susceptible or resistant, had intermediate resistance, according to HI scores at 21 dpi. Resistance conferred by subclones Kpn1.2 and Avr2.1, or Xba1.1 and Sal1.2, were different according to M-W *U* test on rank-ordered T_1_, two-tailed *p* = 0.0011, or 0.0008, respectively. (C) Fractions of *n* FOM-infected (left) or FOC-infected (right) progeny of F_1_ backcross (1A3+Kpn1.2×Ty-0)×Ty-0 with (+) and without (−) copies of *RFO1-C* and *tRFO2* had the lowest, middle or highest third of ranks, according HI scores from four time points. FOC-infected plants with (+) and without (−) *tRFO2* had similar median ranks, according to M-W *U* test (two-tailed *p* = 0.72).

We first confirmed the association of *RFO2* with resistance by testing the resistance of progeny of a new cross, in which *RFO1* and *RFO2* were the only *RFO* QTLs segregating. *RFO1* was included in the cross because previous analysis suggested that the Col-0 allele of *RFO1* (*RFO1-C*) enhances resistance conferred by the Col-0 allele of *RFO2* (*RFO2-C*) [9]. One parent of the cross, plant 4D2, was heterozygous at *RFO2* and homozygous Ty-0 at the remaining *RFO* loci, including *RFO1* (*RFO1-T/T RFO2-C*/*T*). The other parent was the near isogenic line 1A3, which has a small chromosomal region around *RFO1-C* introgressed into the Ty-0 genetic background (*RFO1-C*/*C RFO2-T*/*T*). Among the resulting progeny, plants that inherited *RFO2-C* (*RFO1-C*/*T RFO2-C*/*T*) were more resistant than plants that did not inherit *RFO2-C* (*RFO1-C*/*T RFO2-T*/*T*), which still exhibited more resistance than Ty-0 presumably because all progeny were *RFO1-C/T* (Figure 1A). Also, as previously observed, *RFO1-C* enhanced resistance conferred by *RFO2-C*. Both the self progeny of *RFO1-T/T RFO2-C*/*T* (4D2), which segregated for *RFO2-C* but lacked *RFO1-C*, and Ty-0, which lacked both *RFO2-C* and *RFO1-C*, exhibited similar severe symptoms (Figure 1A). Thus, *RFO1-C* was included in crosses to map *RFO2*.

Using genetic linkage analysis, we mapped *RFO2* to an interval, corresponding to less than 258 kilobasepairs (kb) and fewer than 68 genes, as described in Materials and Methods, which suggested that *RFO2* is the effect of a single gene.

To clone the *RFO2* gene sequence, we tested whether Col-0 genomic sequence in the final *RFO2* interval could enhance the resistance of *RFO1-C*/*C RFO2-T*/*T* (1A3). In total, 19 genomic subclones were stably introduced to line 1A3 using *Agrobacterium tumefaciens*-mediated transformation, and just two subclones enhanced the resistance of 1A3 (Figure S1). Independent kanamycin-resistant T_1_ transformants of subclone Kpn1.2 expressed more resistance to FOM than T_1_ transformants of subclone Avr2.1; and, similarly, T_1_ transformants of Sal1.2 were more resistant than T_1_ transformants of Xba1.1 (Figure 1B).

**Figure 2:**
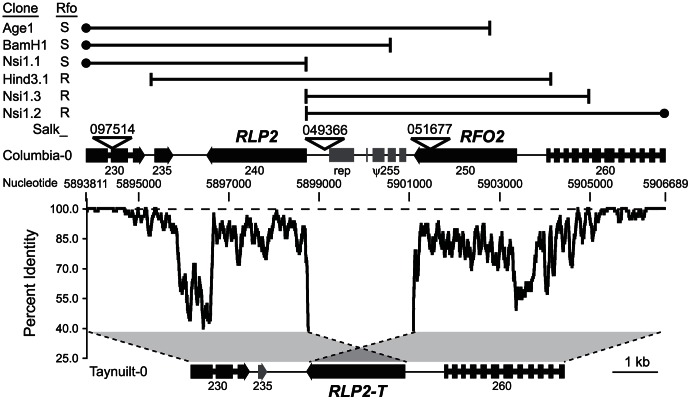
*RFO2* region is highly diverged in Col-0 and Ty-0. Grey regions between dotted lines depict alignment of most of the longer Col-0 sequence at the *RFO2* locus on chromosome 1 with the shorter sequence in Ty-0. At each nucleotide position in Col-0, the percent identity of 37-nucleotides upstream and downstream in the aligned Col-0 and Ty-0 sequences is graphed. Dashed line is at 100 percent identity. Genes and intergenic sequences in the *RFO2* region of Col-0 and Ty-0 are depicted above and below, respectively, at scale (see 1 kb at lower right). Arrowheads indicate orientation of genes, wide at exons and narrow at introns. TAIR-annotated genes (filled) are labeled with last three digits of gene number, after ‘At1g17’, and repetitive element (rep) and noncoding degenerate transcription unit (ψ255) are half-filled. Salk identifiers label inverted triangles pointing to T-DNA insertion sites. Endpoints (vertical lines) and extent (horizontal lines) of Col-0 genomic clones are mapped in the *RFO2* region of Col-0 (above); and, filled circles indicate that cloned sequence extends outside the *RFO2* region. Rfo phenotypes, conferred by genomic clones in transformed 1A3, are listed to the left: either enhanced resistance (R) or no added resistance and susceptible (S).

In the two positive genomic subclones, Sal1.2 and Kpn1.2, there were 13.8 kb of overlapping sequence. This overlapping sequence was further subcloned as six restriction fragments (that are mapped in Figure 2 and Figure S1). Only 1A3 transformants harboring constructs Hind3.1, Nsi1.2 and Nsi1.3 that include gene At1g17250 showed enhanced resistance. Meanwhile, 1A3 transformants, harboring subclones without full-length At1g17250, namely Age1, BamH1 and Nsi1.1, were similarly affected by FOM infection as the untransformed line 1A3.

Many Sal1.2 and Kpn1.2 transgenic lines exhibited unexpectedly strong resistance as compared to the modest *RFO1-C*-dependent resistance expressed by *RFO2* in Col-0 and Ty-0 recombinants. Analysis of a cross, in which both *RFO1-C* and the putative *RFO2-C* transgene (*tRFO2*) were segregating, confirmed that resistance conferred by *tRFO2* was in fact independent of *RFO1-C* and stronger than the resistance of *RFO1-C*. A Kpn1.2 transgenic line (1A3+*tRFO2*) and Ty-0 were crossed, and the resulting F_1_ dihybrid *RFO1-C*/*T tRFO2-*(*+*/*−*) was then backcrossed to Ty-0 to yield F_1_BC progeny, (i) without *RFO1-C* and *tRFO2*, (ii) with *RFO1-C* only, (iii) with *tRFO2* only, or (iv) with both *RFO1-C* and *tRFO2*, in a ratio of 1∶1∶1∶1 that is expected for independent assortment of *RFO1* and *tRFO2* (Figure 1C). Plants with *tRFO2* were more resistant to FOM with or without *RFO1-C*; and, plants with *tRFO2* only were substantially more resistant than plants with *RFO1-C* only (Figure 1C). When F_1_BC progeny were infected with FOC instead, plants with *tRFO2* were no more resistant than plants without *tRFO2* (Figure 1C). Thus, the strong resistance of *tRFO2* was specific for FOM.

**Figure 3:**
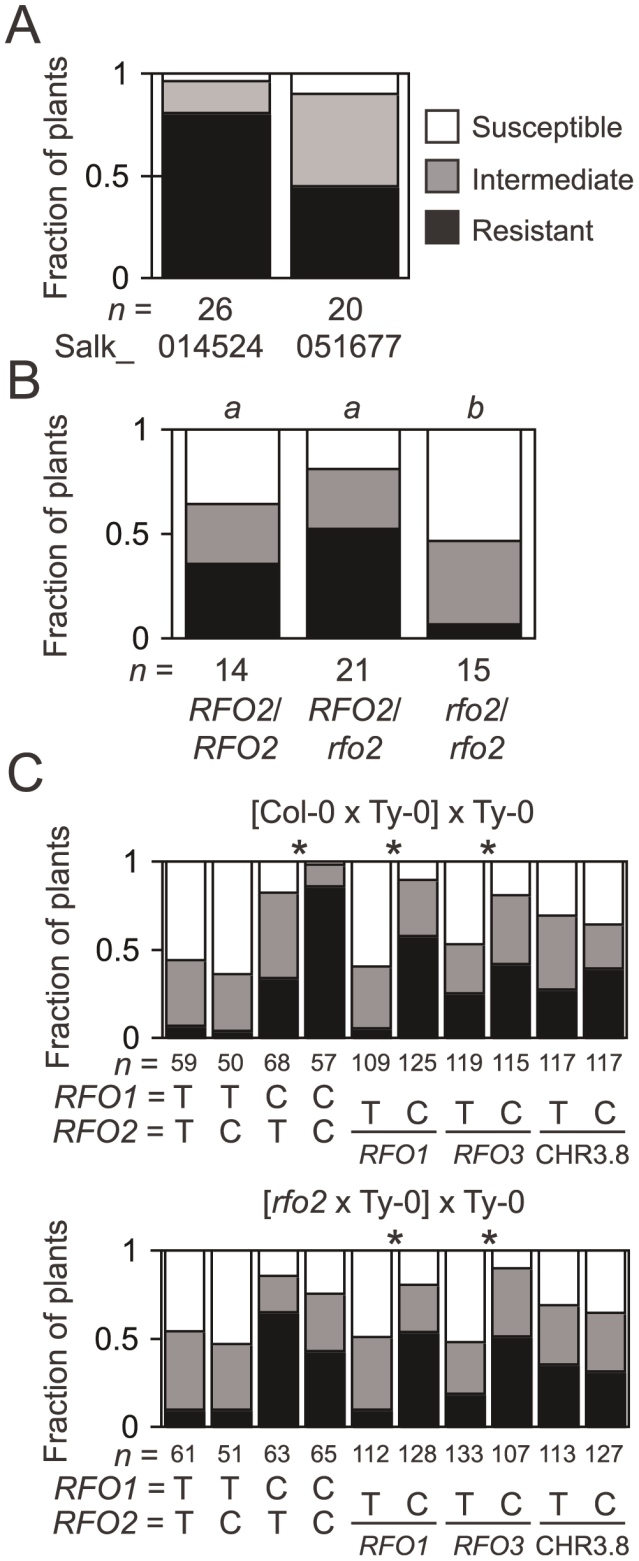
T-DNA insertion allele *rfo2* abolishes *RFO2* QTL. (A) Fractions of *n* F_1_ hybrids of Ty-0 and Salk_014524 (At1g17200) or Salk_051677 (At1g17250) were susceptible or resistant or had intermediate resistance, according to HI scores at 21 dpi. Median ranks of the two F_1_ hybrids are dissimilar, according to M-W *U* test (two-tailed *p* = 0.021). (B) Fractions of *n* self progeny of *rfo1 RFO2*/*rfo2*, either *RFO2*/*RFO2*, *RFO2*/*rfo2* or *rfo2*/*rfo2*, had the lowest, middle or highest third of ranks at 21 dpi. Same italicized letters above genotypes indicates that median ranks were similar, according to M-W *U* test (two-tailed *p*>0.05). (C) Genotypes of 234 F_1_BC progeny of original cross (Col-0×Ty0)×Ty-0 (top) and 240 F_1_BC progeny from new cross (*rfo2*×Ty0)×Ty-0 are either Ty-0/Ty-0 (T) or Col-0/Ty-0 (C), at *RFO1-*, *RFO2-* and *RFO3-*linked markers and marker CHR3.8 that is not linked to a *RFO* QTL. Fractions of *n* FOM-infected plants with the lowest, middle or highest third of ranks. Asterisks indicate that alternative genotypes C and T had dissimilar median ranks, according to M-W *U* test (two-tailed *p*<0.01).

Because multiple *RFO* loci contribute to the complete resistance of Col-0, we anticipated that loss-of-function in *RFO2* alone might not exhibit loss of resistance in the Col-0 genetic background. Indeed lines homozygous for T-DNA insertions in or adjacent to candidate genes in the *RFO2* region were strongly resistant to FOM –see Materials and Methods for details. However, when four insertion lines were crossed to Ty-0, which halved the genetic contribution of Col-0, F_1_ hybrids could develop obvious wilt symptoms. The F_1_ hybrids of Salk_051677, in particular, were especially susceptible, and a majority of these F_1_ hybrids expressed symptoms, while only 20 percent of F_1_ hybrids of Salk 140524 were similarly affected (Figure 3A). T-DNA insertion in Salk_051677 interrupts At1g17250, the same gene that correlated with enhanced resistance in 1A3 transformants, whereas insertion in Salk_140524 interrupts At1g17200, a gene outside the overlapping sequence in Kpn1.2 and Sal1.2. Salk_051677, which was previously named *Atrlp3-1* without a reported phenotype, was renamed *rfo2* [31].

Although plants with genotype *RFO1 rfo2* exhibited strong resistance to FOM, as mentioned above, *rfo2* did enhance susceptibility of *rfo1* in the double mutant *rfo1 rfo2*, which was also more susceptible than the *rfo1 RFO2*/*rfo2* heterozygote (Figure 3B). In the Col-0 genetic background, *RFO2* expressed resistance in the absence of *RFO1* even though resistance conferred by *RFO2* showed dependence on *RFO1* in the original mapping cross between Col-0 and Ty-0 used to define *RFO* QTLs (Figure 3C) [9].

In theory, *RFO2* might correspond to more than one gene because we discovered *RFO2* as a QTL [9]. To address whether At1g17250 alone accounts for the *RFO2* QTL, we examined the segregation of resistance in a comparable (*rfo2*×Ty-0)×Ty-0 mapping population. This new population was similar to our original mapping population with the exception that *rfo2* replaced wild type as the Col-0 parent. Specifically, we crossed *rfo2* and Ty-0 and then backcrossed the resulting F_1_ hybrid to Ty-0. As expected, Col-0/Ty-0 heterozygotes and Ty-0/Ty-0 homozygotes appeared in roughly equal proportion with all tested markers (Figure 3C). DNA markers linked to *RFO1* and *RFO3*, which is a third *RFO* QTL previously detected on chromosome 3 [9], were associated with resistance in both the new and original populations. In contrast, *RFO2-C* showed significant correlation with resistance only in the original population (Figure 3C). In the (*rfo2*×Ty-0)×Ty-0 population, resistance at *RFO2* instead had a modest correlation with Ty-0 homozygotes (*RFO2-T/T*).

### *RFO2* inhibits FOM infection in roots

**Figure 4:**
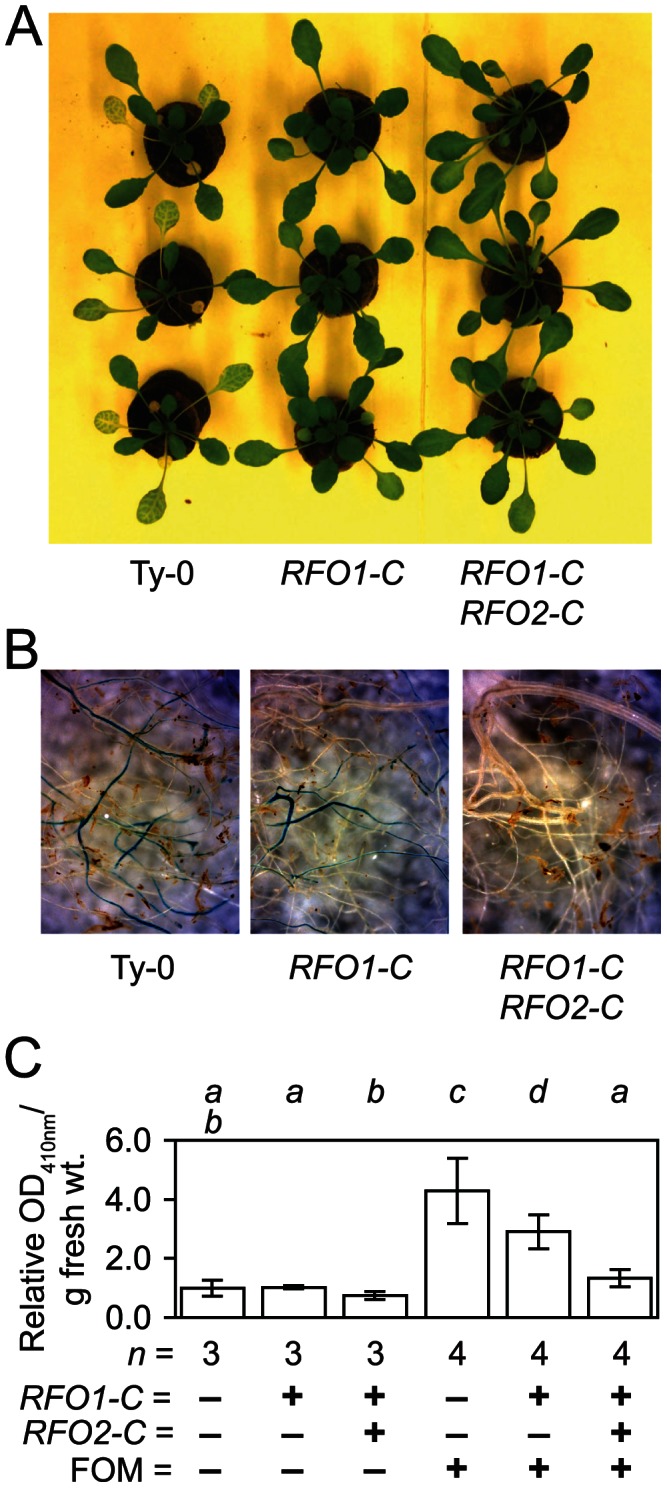
*RFO1-C* and *RFO2-C* restrict infection in roots. All plants have the genetic background of Ty-0, which lacks both *RFO1-C* (−) and *RFO2-C* (−). Line 1A3 and 1A3+*tRFO2* also have *RFO1-C* (+), and only line 1A3+*tRFO2* has *RFO2-C* (+). Plants of each genotype (*n* = 24) were rank-ordered from most susceptible to most resistant, and plants with median or middle ranks are shown and analyzed. (A) Three representative FOM-infected plants of each genotype are shown at 12 dpi. (B) Roots of FOM-infected plants were stained with X-Ara. (C) Relative *Fusarium*-derived ABF activity in FOM- (+) and mock- (−) infected roots, in terms of absorbance (OD_410 nm_) of 4-nitrophenol formed after 16-hr incubation of whole roots, harvested from (*n*) mock-infected (−) or FOM-infected (+) plants, with NP-Ara at 10 dpi. Values are adjusted to set mean value of mock-infected Ty-0 roots equal to one. Error bars are confidence interval of the mean (α = 0.05). Different italicized letters indicate that means are dissimilar, according to Student's *t*-test (*p*<0.05).

Up to now, we equated susceptibility with symptom severity in the above ground foliage, where little if any FOM would be present until late in infection [28]. Possibly, quantitative resistance could reflect reduced symptoms in the expressive phase of infection rather than reduced fungal infection in the below ground roots [32]. To distinguish between these possibilities, we compared the effect of *RFO1-C* and *RFO2-C* on symptoms in shoots and FOM infection in roots. At 12 dpi, *RFO1-C RFO2-C* (1A3+*tRFO2*) exhibited only modest stunting while Ty-0 plants, without the benefit of either *RFO1-C* or *RFO2-C*, were severely stunted, and older leaves were yellowing (Figure 4A). Meanwhile, *RFO1-C* (1A3) developed symptoms that were intermediate to those in Ty-0 and *RFO1-C RFO2-C*. *In situ* staining with X-Ara reports *F. oxysporum* infection as a blue precipitate because *F. oxysporum*, and not *Arabidopsis*, expresses detectable arabinofuranosidase (ABF) activity [28]. Blue staining was stronger and more prevalent in roots of *RFO1-C* than roots of *RFO1-C RFO2-C* while roots of Ty-0 showed the most extensive staining (Figure 4B); and, uninfected roots of all genotypes remained unstained. The observed differences in X-Ara staining were corroborated by quantifying the accumulation of soluble yellow 4-nitrophenol when roots were incubated with a second substrate of ABF, NP-Ara (Figure 4C).

### *RFO2* corresponds to diversity in *PSY1R*-related RLP genes

In prior surveys of RLP genes in the Col-0 reference genome, At1g17240 and the neighboring gene *RFO2*/At1g17250 were identified as a tandem pair of highly-related receptor-like protein (RLP) genes and generically named *RLP2* and *RLP3*, respectively [31], [33]. The primary structure of RFO2 is similar to previously characterized RLPs and is comprised of seven domains (Figure S2) [34]: A signal peptide (domains A), four extracellular domains (B through E), a transmembrane domain (F) and a short cytoplasmic tail of nine amino acids (domain G). Most of RFO2 is extracellular and is composed of 23 extracellular leucine-rich repeats (eLRRs, domain C), which are capped at amino-terminal and carboxy-terminal ends by domains B and D, respectively. An acidic domain E joins the extracellular domains to the transmembrane domain. Also, a loop out sequence interrupts the 19th eLRR in domain C.

Genomic sequence in the chromosomal region around *RFO2* is highly diverged in Col-0 and Ty-0. In order to characterize the susceptible *RFO2-T* allele, we obtained an 8,311 bp sequence that spans the *RFO2* region in Ty-0 using PCR-sequencing. According to BlastN search of the Col-0 reference genome, the best match for the Ty-0 sequence extended across a 12,878 bp interval that included sequence within and between annotated genes At1g17230 and At1g17260, as depicted in Figure 2. In the shorter Ty-0 sequence, a single RLP gene (*RLP2-T*) was oriented on the chromosome in the same direction as the head-to-tail pair of *RFO2* and *RLP2* in Col-0 (Figure 2). Sequence predicted to be intergenic retained remarkably low nucleotide identity in the two accessions, and intergenic sequence between *RFO2* and *RLP2* could not be aligned to any Ty-0 sequence. Thus, the Col-0 and Ty-0 variants of *RFO2* appeared to be ancestral variation in *A. thaliana*.

The alignment of coding sequences in the single exons of the three RLP genes at the *RFO2* locus showed that *RLP2* and *RLP2-T* were more related to each other than *RFO2*. Specifically, the 1,956-nucleotide sequence starting at the 5′ end of *RLP2-T* shared more identity with *RLP2* (88 percent) than *RFO2* (82 percent). However, a shorter 136 bp sequence at the 3′ end of *RLP2-T* shared more identity with *RFO2* (73.5 percent) than *RLP2* (55 percent). Interestingly, *RLP2* shared most identity (92 percent) with the full-length ortholog *AlRLP2* from *Arabidopsis lyrata* than even a partially aligned *RLP2-T*.

From BlastP searches of the *Arabidopsis* genome database, we learned that RFO2-related RLPs share conspicuous similarity with the extracellular regions of the *Arabidopsis* RLK PSY1R that perceives the small post-translationally modified tyrosine-sulfated peptide hormone PSY1 involved in cell division and expansion [29]. Specifically, alignment of B and C domains of either RFO2 or RLP2 and PSY1R showed that 74 or 80 percent of residues in the eLRRs, respectively, were identical (Figure S3). Remarkably, RFO2 and RLP2 were more similar to PSY1R than they were to each other as just 73 percent of residues were identical in the alignment of B and C domains of RFO2 and RLP2 (Figure S3). However, outside of the eLRRs, there was little or no sequence conservation between RLK and either RLP, and PSY1R poorly aligned to domains D through G of RFO2 or RLP2 (Figure S4).

### The carboxy-end of *RFO2* specifies resistance to FOM

**Figure 5:**
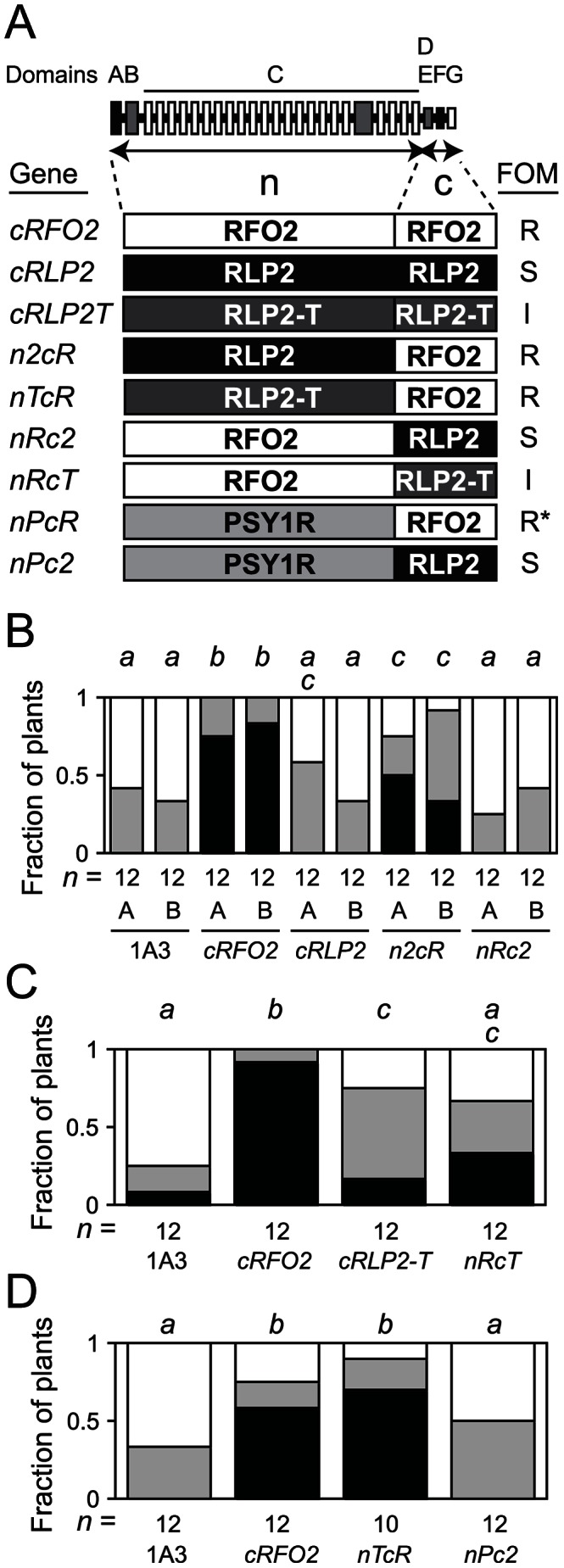
Constitutively-expressed and chimeric RLP genes. (A) Domain structure of *RFO2*-related RLPs is shown as a series of boxes. From left to right, signal/anchor peptide (A, filled), N-cap (B, half-filled), eLRR subdomains (open) and loop out subdomain (half-filled) of domain C, C-cap/acidic domain (D/E, half-filled), transmembrane domain (F, filled) and cytoplasmic tail (G, open). Amino-terminal domains A through C (n) and carboxy-terminal domains D through G (c) of RFO2-related RLPs RFO2 (R), RLP2 (2) and RLP2-T (T) and the n-domain of PSY1R (P) are combined in the constitutive expression vector ORE-E3 to make the three original and six chimeric RLP transgenes as listed with names to the left. On the right, Rfo phenotypes of listed RLP transgenes: strong resistance (R), intermediate resistance (I) or no effect on susceptibility of line 1A3 (S) to FOM infection. *nPcR* confers strong resistance to FOC, FOM and FOR (*). (B) Experiments A and B were performed with independent T_2_ lines, and plants in separate experiments were rank-ordered at 24 dpi. Fractions of *n* FOM-infected untransformed 1A3 or Ppt-resistant 1A3 T_2_, transformed with the constitutively expressed RLP transgenes (above), with the lowest, middle, highest third of ranks were susceptible (open), had intermediate resistance (half-filled) or were resistant (filled). Same italicized letters above columns indicates that median ranks were similar according to M-W *U* test, *p*>0.05. (C and D) Fractions of *n* FOM-infected untransformed 1A3 or Ppt-resistant 1A3 T_2_, transformed with constitutively-expressed RLP transgenes, with the lowest, middle or highest third of ranks at 24 dpi were susceptible (open), had intermediate resistance (half-filled) or were resistant (filled).

By using the same constitutive promoter to express *RFO2*, *RLP2* and *RLP2-T*, we tested whether differences in transcription could explain why *RFO2* conferred resistance while its homologs *RLP2* and *RLP2-T* did not. Coding sequences of the three RLP genes were fused downstream of the constitutive promoter *ENTCUP2*, and these promoter-gene fusions were introduced to line 1A3 by stable genetic transformation [35], [36]. Phosphinothricin (Ppt)-resistant T_1_ transformants of 1A3 harboring the three promoter-gene fusions exhibited the same wild-type appearance as the untransformed line 1A3. Independent T_2_ lines harboring constitutively-expressed *RFO2* (*cRFO2*) exhibited strong resistance to FOM while, in the same infection assays, independent T_2_ lines harboring constitutively-expressed *RLP2* (*cRLP2*) appeared similar to the untransformed parental line 1A3 (Figure 5A and 5B). Meanwhile, a T_2_ line with constitutive expression of *RLP2-T* (*cRLP2-T*) exhibited marginally more resistance than the parental line 1A3, and this resistance was modest in comparison to the resistance conferred by *cRFO2* in the same assay (Figure 5C).

Because the resistance of *RFO2* was not a consequence of differences in promoter expression of the three RLP genes, we next examined whether resistance could be localized to the PSY1R-related amino-terminal (n-) domains (A through C) or the more diverged carboxy-terminal (c-) domains (D through G) of the three RLPs. Coding sequences for the n- and c-domains were recombined to make chimeric RLP genes. A unique *Spe*I restriction site was introduced as a silent mutation in coding sequence of *cRFO2*, *cRLP2* and *cRLP2-T* that joins domains C and D (Figure S4). Sequences on either side of the *Spe*I site, coding for n- or c-domains, were swapped (as depicted in Figure 5A), and the resulting chimeric RLP genes were stably introduced to line 1A3 by genetic transformation. Ppt-resistant T_2_ plants harboring in vitro constructed chimeric genes with the n-domains of either RLP2 or RLP2-T and the c-domains of RFO2 in Figure 5B or 5D, respectively, expressed strong resistance to FOM; henceforth, these chimeric genes are referred to as n2cR and nTcR, respectively. Meanwhile, Ppt-resistant T_2_ plants harboring transgenes with the reciprocal exchanges, coding sequence of the n-domains of RFO2 fused to sequence of the c-domains of RLP2 (*nRc2*) or RLP2-T (*nRcT*), expressed resistance to FOM similar to the untransformed parental line 1A3 and *cRLP2* (in Figure 5B) or the original *cRLP2-T* (in Figure 5C), respectively. Thus, the shorter, less conserved sequence of the c-domains of RFO2 specified resistance to FOM, and the function of the conserved eLRRs of the three RLP homologs was roughly equivalent for resistance.

### *PSY1R*, *PSKR1*, and *PSKR2* promote susceptibility

The sequence conservation of eLRRs in RFO2 and PSY1R prompted us to examine wilt disease progression in loss-of-function mutant *psy1r* [29]. The function of *PSY1R* overlaps with the function of two closely related RLK genes *PSKR1* and *PSKR2* that perceive the tyrosine-sulfated peptide PSK [29], [30]. PSK accumulates in cell culture medium and is a key factor permitting the dedifferentiation and redifferentiation of plant cells in culture [37]. Signaling by peptides PSY1 and PSK negatively regulates stress response and senescence, and addition of PSK and PSY1 to agar medium promotes elongation of roots of *Arabidopsis* seedlings [29], [30], [37]. The functional overlap of PSY1 and PSK signaling prompted us to test the infection of *pskr1* and *pskr2* as well as *psy1r*.

**Figure 6:**
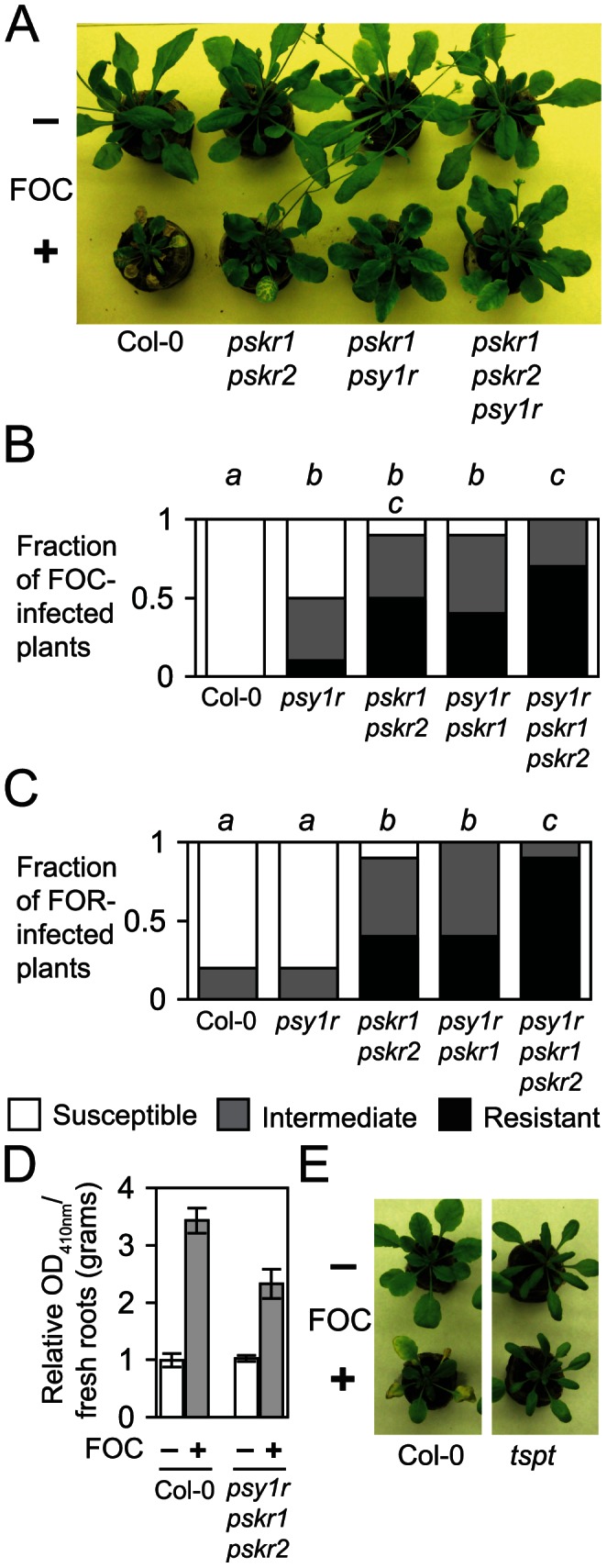
PSY1 and PSK promotes susceptibility to Fusarium wilt. (A) Representative FOC-infected (+) or mock-infected (−) wild type (Col-0) and mutants having median HI scores (*n* = 6 for each genotype) are shown at 24 dpi. (B) Fraction of FOC-infected or (C) FOR-infected plants (*n* = 10 for each genotype) with the lowest, middle, highest third of ranks at 18 dpi. Different italicized letters indicates that median ranks of genotypes were dissimilar (M-W *U* test, *p*<0.05). (D) Relative *Fusarium*-derived ABF activity in FOC- (+) and mock- (−) infected roots, in terms of absorbance (OD_410 nm_) of 4-nitrophenol formed after 20-hr incubation with NP-Ara, was different at 10 dpi, according to Student's *t*-test (*n* = 4; two-tailed *p* = 0.0001). Error bars are confidence interval of the mean (*α* = 0.05). (E) Representative FOC-infected (+) or mock-infected (−) wild type (Col-0) and *tpst* having median HI scores (*n* = 10 for each genotype) are shown at 18 dpi.

When plants that are insensitive to PSY1 (*psy1r*), insensitive to PSK (double mutant *pskr1 pskr2*) or insensitive to both PSY1 and PSK (*psy1r pskr1* double mutant and *psy1r pskr1 pskr2* triple mutant) were infected with FOM, all mutants were completely resistant. However, the receptor mutants have the Col-0 genetic background, and Col-0 is already completely resistant to FOM. When plants were instead infected with FOC (Figure 6A and 6B) or FOR (Figure 6C), two formae speciales to which Col-0 normally expresses incomplete resistance, mutants were noticeably more resistant than wild type. The triple mutant that is insensitive to both PSK and PSY1 peptides showed the strongest suppression of disease symptoms while mutants that are insensitive to either PSK or PSY1 showed a more modest suppression of disease. X-Ara staining of FOC-infected roots of the triple mutant suggested that initial infection of root tips was normal but indicated that subsequent infection of xylem by *F. oxysporum* was suppressed. We quantified the diminished fungal infection in roots using NP-Ara (Figure 6D), and two-fold less *F. oxysporum*-derived ABF activity was measured in roots of the triple mutant than wild type.

We next examined whether perception of endogenous PSY1 and PSK peptides was critical for the susceptibility that peptide hormone receptor genes expressed in wild type. *Arabidopsis* has a single tyrosyl-protein sulfotransferase gene *TPST*, and *tpst* produces only unsulfated and inactive PSY1 and PSK peptides [38], [39]. FOC-infected *tpst* expressed strong resistance that was comparable to the enhanced resistance of *psy1r pskr1 pskr2* (Figure 6E), which suggested that endogenous peptide signaling suppressed resistance to *F. oxysporum*.

### Resistance of a PSY1R-RFO2 chimeric RLP

Because the eLRRs of RFO2 and PSY1R have remarkable similarity, we examined whether their eLRRs also share a common, interchangeable function by reciprocally swapping their homologous n-domains (Figure S3) and testing the resulting chimeric RLP and RLK genes for function in place of *RFO2* and *PSY1R*, respectively. On the one hand, the chimeric RLP gene *nPcR* was the fusion of sequence coding for the extracellular n-domains of *PSY1R* (including domains A through C) to sequence coding for the membrane proximal c-domains of *RFO2* (including domains D through G, as depicted in Figure 5A). On the other hand, the chimeric RLK gene *nRcP* was the fusion of sequences coding for n-domains of *RFO2* and c-domains of *PSY1R*.

**Figure 7:**
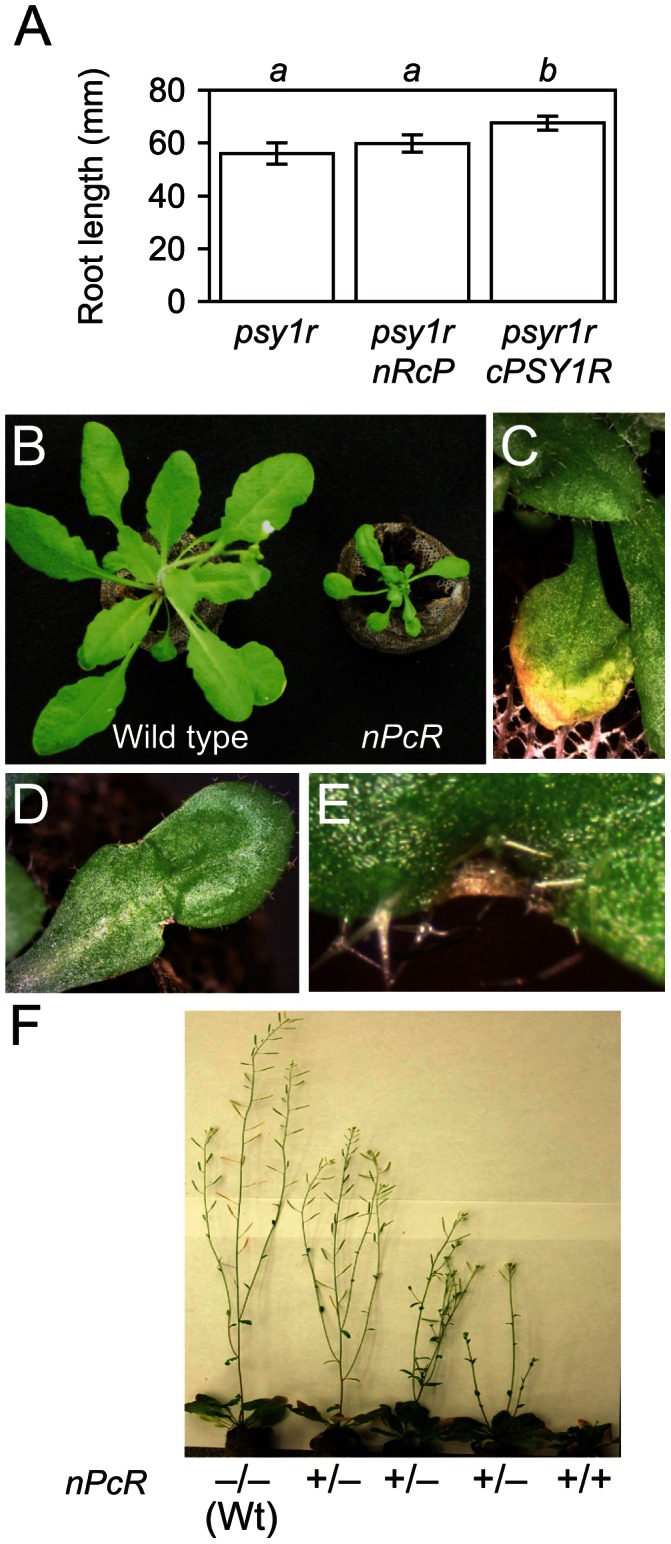
Phenotypes of *cRFO2* and *cPSY1R* chimeric RLP and RLK genes. (A) Mean root length of *psy1r*, *nRcP psy1r* and *cPSY1R psy1r* after two weeks of growth on vertical PN agar plates. Error bars are the confidence interval of the mean (*α* = 0.05). Same italicized letter indicates that means were similar, according to Student's *t* test (*p*>0.05; for all genotypes, *n* = 15). Similar results were reproduced with independent T_2_ lines. Typical phenotypes of representative *nPcR* transgenic line 1E9: (B) smaller rosette leaves of four-week-old nPcR homozygote (right) as compared to leaves of its wild-type Col-0 parent (left); (C) senescence of leaves (before senescence of leaves of comparable wild type); (D) malformed, misshapened rosette leaf; and, (E) macroscopic necrotic lesion at leaf margin. (F) Severity of pleiotropy cosegregated with herbicide resistance marker linked to *nPcR* in self progeny of 1E9 hemizygote: Progeny of plants with normal appearance (wild type, Wt) were all Ppt-sensitive (−/−), progeny of the most affected were all Ppt-resistant (+/+), and Ppt resistance and sensitivity segregated (+/−) in progeny of plants with intermediate phenotypes.

Even though amino acid similarity in n-domains of RFO2 and PSY1R is comparable to the similarity in functionally equivalent n-domains of RFO2, RLP2 and RLP2-T (Figure S3), n-domains in RFO2 and PSY1R proved to have dissimilar function. For the sake of comparison, we also fused full-length coding sequence of *PSY1R* downstream of the constitutive promoter *ENTCUP2* to make *cPSY1R*. Endogenous PSY1 promotes the growth of roots, and the PSY1-insensitive roots of *psy1r* are shorter than wild-type roots on agar plates [29]. Stable transformation of *psy1r* with *cPSY1R* appreciably enhanced root growth while roots of independent transformants of *psy1r* harboring the chimeric RLK gene *nRcP* were not significantly longer than untransformed *psy1r* roots (Figure 7A).

Unexpectedly, most Ppt-resistant T_1_ transformants harboring the chimeric RLP gene *nPcR* exhibited obvious pleiotropy. Phenotypes of *nPcR* were reminiscent of the constitutive resistance that is displayed by activated resistance genes in mutants or the autoimmunity of hybrid necrosis, resulting from crosses between particular *Arabidopsis* accessions [40]–[43]; and, *nPcR* transformants of Col-0 or line 1A3 were similarly affected. Indeed, *nPcR* conferred complete resistance to FOC, FOM and FOR as *nPcR* plants in *F. oxysporum*-infected and mock-infected soil were indistinguishable; and, X-Ara staining detected no vascular infection in *nPcR* roots at 12 dpi. *nPcR* transformants had smaller (Figure 7B), often misshapen rosette leaves (Figure 7D) that had macroscopic lesions (Figure 7E) and were prone to senesce before wild-type leaves (Figure 7C). *nPcR* inflorescences were stunted and occasionally arrested by necrosis at their apices. *nPcR* pleiotropy was dose dependent as phenotypes were consistently less and more severe among *nPcR* hemizygotes and *nPcR* homozygotes in the same transgenic line, respectively (Figure 7F); and, phenotypes were more and less severe at high (30°C) and low (22°C) temperatures, respectively. We never observed similar phenotypes in transformants harboring other RLP constructs, including *cRFO2*.

To test whether the c-domains of RFO2 were critical for expresfsion of *nPcR*-related phenotypes, coding sequences of n-domains of PSY1R and c-domains of RLP2 were fused in the chimeric RLP gene *nPc2* (Figure 5A). Ppt-resistant T_1_ plants harboring *nPc2* had wild-type appearance, and T_2_ plants showed no enhanced resistance to FOM (Figure 5D). Thus, both strong resistance to FOM and visible pleiotropy required the c-domains of RFO2.

Considering that RLPs and RLKs may self-associate as dimers or in oligomeric complexes, we tested whether *RFO2* and *PSY1R* contributed to the pleiotropy of *nPcR* by examining the effect of *rfo2* and *psy1r* on *nPcR* [44], [45]. In a representative *nPcR* transgenic line 1E9, Ppt-resistance and pleiotropy cosegregated as a single locus. Pure-breeding 1E9 (*nPcR*) was crossed to both *rfo2* and the double mutant *pskr2 psy1r*, and self-crosses of the resulting F_1_ plants generated F_2_ progeny. Among the Ppt-resistant F_2_ of cross *nPcR*×*rfo2*, the three possible genotypes of *RFO2* segregated with the expected ratio of 1∶2∶1 (Table 1). When F_2_ were rank-ordered by size, the median ranks of *nPcR*/*– rfo2* and *nPcR*/*– RFO2* were comparable (two-tailed *p* = 0.52, using Mann-Whitney *U* test), and thus *rfo2* had no effect on the small stature of *nPcR*. Among the Ppt-resistant F_2_ of cross *nPcR*×*pskr2 psy1r*, *PSKR2* segregated with the expected ratio of 1∶2∶1 (Table 1). However, among the 48 Ppt-resistant (*nPcR*/*–*) F_2_, there were no *psy1r* homozygotes, and the observed segregation of *PSY1R* significantly deviated from the expected ratio of 1∶2∶1 (*p* = 0.0003, Table 1); in fact, numbers of wild-type homozygotes (*PSY1R*/*PSYR1*) and *PSY1R*/*psy1r* heterozygotes approximated the ratio (1∶2∶0) expected for a recessive lethal condition (*p* = 0.54). To confirm that *PSY1R* and *nPcR* were unlinked and *psy1r* homozygotes were viable in the absence of *nPcR*, we genotyped 32 Ppt-sensitive F_2_ and obtained the expected ratio of 1∶2∶1 for genotypes at *PSY1R* (Table 1). Thus, viability of *nPcR*-expressing plants required *PSY1R*.

**Table 1:** F_2_ segregation of mutants in crosses with transgene *nPcR*.

Parental cross	*nPcR* a	Gene^b^	*N* c	Genotype of F_2_						*p* d
				Observed^e^			Expected^f^			
				W	H	M	W	H	M	
*nPcR*×*rfo2*	+	*rfo2*	51	14	22	15	13	25	13	0.69
*nPcR*×*pskr2 psyr1*	+	*pskr2*	45	12	23	10	11	23	11	0.91
	+	*psy1r*	48	18	30	0	12	24	12	**0.0003**
	−	*psy1r*	32	6	20	6	8	16	8	0.37
*nPcR*×*tspt*	+	*tpst*	19	7	12	0	5	9	5	**0.033**
	−	*tpst*	24	6	13	5	6	12	6	0.88

a F_2_ progeny were selected for Ppt resistance (+), which cosegregates with *nPcR*, or sensitivity (−).

b See Materials and Methods for codominant DNA markers used to genotype particular genes.

c Number of F_2_ plants genotyped.

d Probability from chi-squared test that observed genotypes fit expected 1∶2∶1 ratio, derived from random segregation of wild-type and mutant alleles. Degrees freedom = 2. *p* values less than 0.05 are in bold type.

e Observed genotypes were wild-type homozygotes (W), mutant homozygotes (M) and heterozygotes (H).

f Expected monohybrid segregation ratio is 1 wild type : 2 heterozygotes : 1 mutant.

Because PSY1R is the putative receptor of PSY1, we tested whether the viability of *nPcR*-expressing plants also required the presence of active PSY1. As PSY1 is unsulfated and inactive in *tpst*, we crossed *nPcR* and *tpst*. In the self F_2_ progeny of cross *nPcR*×*tpst*, only wild-type homozygotes and *TPST*/*tpst* heterozygotes were identified among 19 herbicide-resistant (*nPcR*/–) F_2_, and their numbers approximated the 1∶2∶0 ratio (*p* = 0.62) and not the 1∶2∶1 ratio (*p* = 0.033), whereas *TPST* genotypes among 24 herbicide-sensitive F_2_ approximated the 1∶2∶1 ratio (*p* = 0.88, Table 1). Thus, *nPcR*-expressing plants depended on the presence of active, sulfated peptides, including PSY1, and the presence of PSY1R.

## Discussion

Phylogenic analysis implicates most of the 90 and 57 RLP gene sequences in the reference genomes of rice and *Arabidopsis*, respectively, in host response to biotic stress [34]. Most RLP genes are members of species-specific clades and (76 percent in rice and 58 percent in *Arabidopsis*) are clustered at loci with two or more related genes [33]. Species-specific genes and gene clustering are also features of the NB-LRR family of resistance genes and imply that lineages of RLP genes are expanding, contracting and diversifying to meet the evolving challenge of infectious disease [46], [47]. Indeed, reverse genetic approaches show that loss-of-function mutations in three *Arabidopsis* RLP genes quantitatively compromise innate immunity to virulent and nonhost pathogens [31], [48], [49].

However, diversity in RLP genes has not been associated with a disease resistance trait in *Arabidopsis* or rice until now. In cultivated species such as tomato, RLP resistance traits are typically the result of interspecific breeding or the inadvertent propagation of loss-of-function polymorphisms [50], [51]. The highly diverged *RFO2* alleles suggest that diversity in RLP genes contributes to quantitative variation in resistance in wild species.

Although monogenic resistance traits are usually associated with NB-LRR genes, several RLP genes confer strong monogenic resistance to specific pathogens as well [52]. The strong pathogen-specific resistance of transgenic *RFO2* is reminiscent of such gene-for-gene resistance. The best-studied RLP genes are in the *Cf* clade and mediate resistance to specific races of the foliar fungal pathogen *Cladosporium fulvum* that express corresponding avirulence genes [50]. Meanwhile, apple *Vfa1* and *Vfa2* confer resistance to five races of the obligate fungal pathogen *Venturia inaequalis*, tomato *LeEix2* confers recognition of an ethylene-inducing xylanase from biocontrol fungus *Trichoderma viride*, oilseed rape *LepR3* confers resistance to *Leptosphaeria maculans* expressing *AvrLm1*, and tomato *Ve1* confers strong resistance to races of *Verticillium* species expressing avirulence gene *Ave1* [53]–[56]. Interestingly, *Ve1* also confers modest quantitative resistance to virulent *F. oxysporum* forma specialis *lycopersici* [53]. Likewise, we presume that *RFO2* perceived an extracellular *Fusarium*-derived signal that was present in FOM infection and absent in FOC infection [57]. However, we cannot discount that FOC infection suppressed *RFO2*'s perception of a signal that was present in all *F. oxysporum* infections. In either case, once induced, *RFO2* was effective against all three crucifer-infecting formae speciales as the constitutive resistance of *nPcR* lacked specificity.

PSY1 and PSK signaling compromised resistance to vascular infection by *F. oxysporum*. Recently, Igarashi et al. reported that *pskr1* (but not *pskr2*) is more resistant to leaf infection by virulent *P. syringae* [58]. *PSY1R*, *PSKR1* and *PSKR2* were identified and characterized for perception of PSY1 and PSK and for the effects that this perception has on root growth, cell proliferation and senescence [29], [30]. Igarashi et al. suggest that PSK signaling directs allocation of resources between energy-intensive processes, toward growth and away from immunity [58]. However, PSK and PSY1 signaling more fully influences the longevity and growth potential of mature differentiated cells [29], and absence of peptide signaling in the triple mutant arguably had a more modest effect on plant mass than wilt resistance (in Figure 6A). Natural resistance traits, such as *RFO1*, *RFO2* and tomato *Immunity* genes, promote resistance to Fusarium wilt by inhibiting infection in the vascular cylinder [2], [28]. Because PSK depresses stress responses in general and immunity in particular, we suspect that peptide signaling depressed the considerable but incomplete resistance of Col-0 to FOC and FOR [9], [37], [58], [59]. The strong resistance of *tpst* suggests that endogenous PSY1 and PSK depressed immunity, though the expression of other proteins with tyrosine sulfation, including root meristem growth factors, is also affected by *tpst* [60]. The strong wilt resistance of the receptor triple mutant does not tell us whether FOC or FOR normally exploits PSY1 and/or PSK signaling to induce susceptibility; however, it does demonstrate that manipulation of even basal signaling would be a fruitful target for pathogen effectors.

Amino acid identity in the eLRRs of RFO2 and PSY1R is conspicuous because RLPs and RLKs usually lack meaningful sequence conservation beyond the structural constraints of the eLRR motif [33]. Premature termination of translation in an RLK gene, such as *Xa21D*, may give rise to a residual RLP-like gene [61]. However, *RFO2* is not simply a truncation of *PSY1R* as the RFO2-related RLPs and PSY1R have little if any sequence conservation outside of the eLRRs (see Figure S4). The regular presence of *PSY1R*-related RLK genes and sporadic distribution of *RFO2*-like RLP genes in plant genomes in the Phytozome v8.0 database presumably reflects the distinct roles of these genes in peptide signaling and defense response, respectively (A.D., unpublished data) [62].

In spite of the relatedness of eLRRs of RFO2 and PSY1R, we failed to connect *RFO2* to a role in PSY1 signaling in normal root growth. PSY1 supplementation enhances root growth, and roots of *psy1r* are shorter than wild-type (Figure 7A) [29]. However, neither transgenic expression (*tRFO2* and *cRFO2*) nor deficiency (*rfo2*) of *RFO2* affected root length; and, we found that root lengths of *psy1r* and *psy1r rfo2* were comparable (Y.S., unpublished data).

*RFO2*'s similarity to *PSY1R* and lack of function in PSY1 signaling are consistent with the decoy model for perception of pathogen effectors [63]. In theory, effectors that target PSY1R might select for a decoy receptor, such as RFO2, that mimics the interaction between effectors and PSY1R but lacks the function that effectors are targeting. Because PSY1 signaling suppressed immunity to *F. oxysporum* infection, the relevant effector would be an agonists or positive regulator of PSYR1. Although how PSY1R perceives PSY1 is unknown, PSK directly bind to the PSK receptor, and a photo-activated analog of PSK preferentially labels the loop out sequence within eLRRs [64].

Considering this decoy model, we were surprised that the c-domains of RFO2 were responsible for resistance to FOM. We anticipated that sequence coding for the n-domains, including eLRRs, would distinguish *RFO2* from homologs (*RLP2* and *RLP2-T*) that failed to confer resistance. Instead, n-domains of RFO2, RLP2 and RLP2-T were functionally equivalent. Contrast this with similar domain-swapping experiments that invariably map recognition of specific *C. fulvum* effectors to sequence in eLRRs of Cf proteins [50]. Interestingly, the critical role for membrane-proximal c-domains of RFO2 is consistent with the observation that *cRLP2-T*, unlike *cRLP2*, expressed some resistance (albeit weaker than *cRFO2*). While *RLP2-T* is generally more related to *RLP2*, the c-domains of RLP2-T and RFO2 are more similar to each other and dissimilar to RLP2 (Figure S4). That RLP2 is a nonfunctional pseudogene could explain the lack of resistance from *RLP2*, just as a loss-of-function polymorphism accounts for the susceptible allele of *Ve1* [51]. However, Wang et al. report that *RLP2* is functional [65]. When the *CLV2* promoter is used to ectopically-expresses *RLP2*, wild-type carpel number and pedicel length is restored to *clv2*. Suppression of *clv2* by *RLP2*, though an abnormal gain of function, clearly shows that *RLP2* can compensate for loss of another RLP gene. Possibly, resistance occurs with *RFO2* and not *RLP2* because defense signaling is engaged by the c-domains of RFO2 and not by the c-domains of RLP2.

The *nPcR* pleiotropy is reminiscent of constitutive activation of resistance in a number of laboratory mutants and transgenic plants [41], [43], and *nPcR* showed complete resistance to infection by FOC, FOM and FOR. However, it should be noted that *nPcR* roots, like *nPcR* shoots, had stunted and irregular growth. Nevertheless, roots of *tir3* are stunted and have irregular growth too, and FOC infection of *tir3* and wild type is similar [28].

The critical role of the c-domains of RFO2 in both the FOM-specific resistance of *cRFO2* and constitutive resistance of *nPcR* suggests that the pleiotropy of *nPcR* is the aberrant, constitutive activation of resistance that FOM normally induces via *RFO2*. When the c-domains in RLP2, which expressed no resistance to FOM, replaced c-domains of RFO2 in *cRFO2* and *nPcR*, the resulting RLPs *nRc2* and *nPc2* expressed neither resistance to FOM nor visible pleiotropy. If pleiotropy were simply the consequence of expressing a truncated PSY1R without a kinase domain, *nPc2* should also express visible pleiotropy.

In an attempt to explain the constitutive resistance of *nPcR*, we recalled that *PSY1R* perceived endogenous PSY1 while *RFO2* appeared insensitive. When the n-domains of RFO2 and PSY1R were swapped, the n-domains of RFO2 appeared insensitive to PSY1 in the chimeric RLK nRcP, which failed to suppress the reduced root growth of *psy1r* (Figure 7A). We reasoned that RFO2 was only activated by an FOM-derived signal, while nPcR was continuously activated by endogenous PSY1. If this hypothesis were correct, simply removing endogenous PSY1 should abolish the pleiotropy of *nPcR*.

Contrary to expectation, absence of active sulfated peptides, including PSY1, (in *tpst*) as well as loss of the PSY1 receptor (in *psy1r*) exacerbated the phenotype of *nPcR*. We imagine that plants with the *tpst nPcR* and *psy1r nPcR* genotypes were not recovered because constitutive resistance had attained a lethal level of expression. This would be consistent with the obvious effect that a two-fold difference in *nPcR* copy number in hemizygotes and transgene homozygotes had on *nPcR* phenotypes (in Figure 7F) [66], [67]. Genetic analysis discounted trivial explanations for why *tpst nPcR* and *psy1r nPcR* were not recovered, such as linkage between the *nPcR* transgene and mutation. Thus, paradoxically, we found that PSY1 was not required to activate resistance via *nPcR*, rather PSY1 signaling was negatively regulating the constitutive resistance of *nPcR*.

We considered the possibility that PSY1 and PSK signaling indirectly suppressed the constitutive resistance of *nPcR* in wild type. Because *psy1r pskr1 pskr2* and *tpst* strongly enhanced resistance to *F. oxysporum* infection, it was possible that an enhanced defense response in *tpst* made the constitutive resistance of *nPcR* lethal. On the other hand, *psy1r* had a much more modest effect on resistance to *F. oxysporum* than the triple receptor mutant or *tpst*, so it seemed remarkable that *psy1r* would have as profound an effect on viability as *tpst*. Nevertheless, if basal signaling of PSY1 and PSK were suppressing the constitutive resistance of *nPcR*, we reasoned that inducing PSK signaling by supplementation with PSK should counteract constitutive resistance and improve growth of *nPcR* seedlings, including roots. According to Igarashi et al., exogenous PSK can suppress elicitor-induced root growth inhibition [58]. However, we found that added PSK failed to have an appreciable effect on the abbreviated root growth of *nPcR* seedlings or improve the appearance of *nPcR* seedlings, even as PSK was able to stimulate the root growth of wild-type and *psy1r* seedlings (Figure S6). Furthermore, *pskr2 nPcR*, which has a partial deficiency in PSK signaling, was recovered from crosses and was indistinguishable from *nPcR* siblings, though both *pskr2* and *psy1r* contributed to resistance to *F. oxysporum* (in Figure 6). Because manipulation of PSK signaling failed to alleviate or exacerbate the pleiotropy of *nPcR*, PSY1 signaling appeared to be intimately associated with negative regulation of *nPcR*.

**Figure 8:**
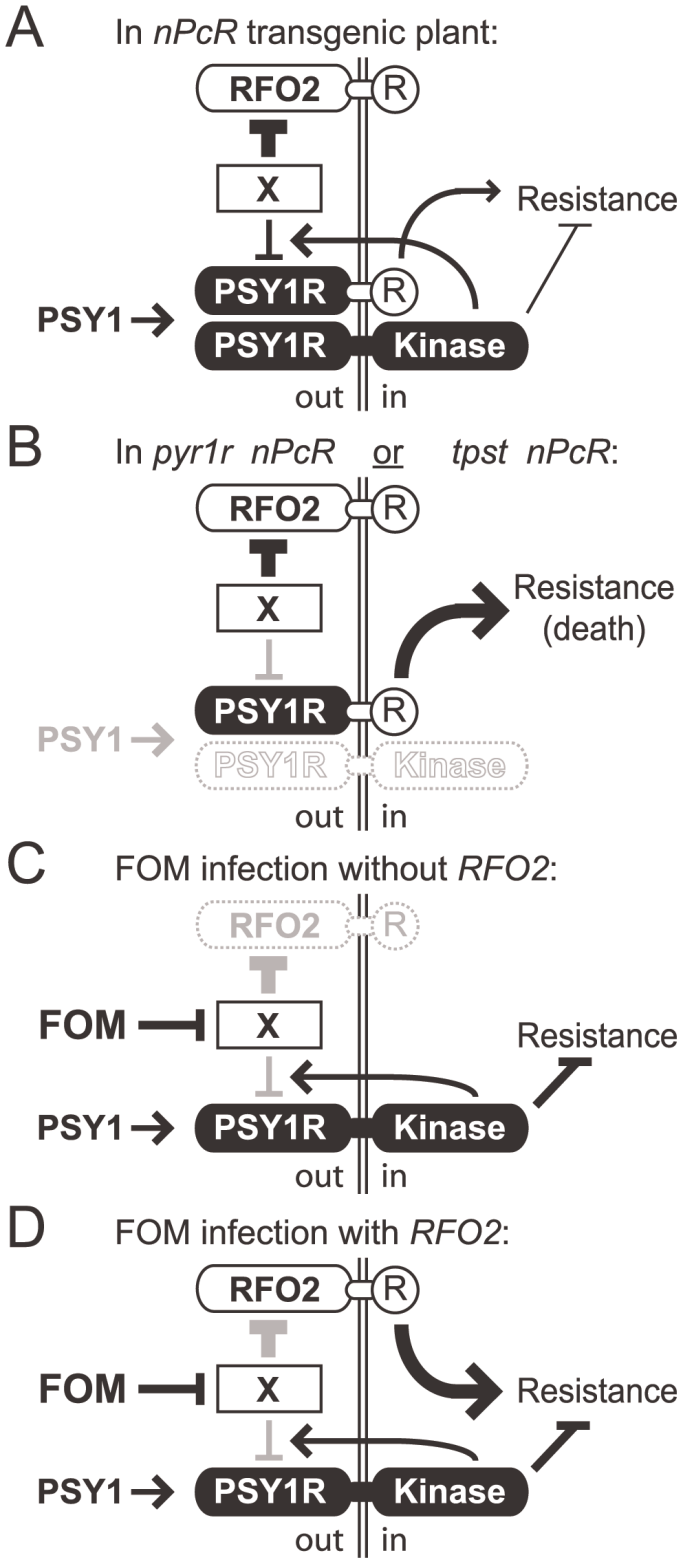
Model for RFO2-mediate resistance. In our model, the extracellular LRRs of PSY1R are activated by PSY1, and the intracellular kinase domain of PSY1-activated PSY1R then (i) tranduces PSY1 signaling, depressing resistance to *F. oxysporum* and (ii) promotes downregulation of PSY1-activated PSYR1. Downregulation has an extracellular component (X) because the eLRRs of PSY1R are presumed to be the target of downregulation in nPcR. RFO2 is a decoy receptor, mimicking the PSY1-activated eLRRs of PSY1R that are competent for downregulation by X. Extracellular (out) and intracellular (in) compartments are on either side of the cell membrane. (A) In *nPcR*, nPcR is incompletely downregulated by PSY1 negative feedback regulation. Consequently, stable expression of the c-domains (R) of RFO2 in nPcR induces resistance constitutively. (B) In *tpst nPcR* or *pyr1r nPcR*, expression of nPcR is further stabilized by loss of all downregulation, by loss of PSY1 activation (in *tpst*) or by loss of PSY1-activated PSY1R (in *psy1r*). Expression of resistance exceeds a lethal threshold without downregulation. Nevertheless, X downregulates RFO2 independent of PSY1 signaling. (C) In infected *rfo2*, FOM inhibits X and downregulation of PSY1-activated PSY1R. Stabilized PSY1-activated PSY1R upregulates PSY1 signaling and exaggerates normal suppression of resistance. s(D) In infected *RFO2*, FOM's inhibition of X also stabilizes RFO2 expression and thereby induces a robust resistance response.

To account for the PSY1-dependent negative regulation of *nPcR*, we hypothesize that an activity-dependent negative feedback mechanism that normally controls the expression of *PSY1R* also controls the expression of *nPcR* (Figure 8A). In this scenario, engagement of PSY1 and PSY1R has two consequences, (i) transduction of PSY1 signaling and (ii) downregulation of PSY1-activated PSY1R. Engagement of PSY1 and nPcR, on the other hand, targets nPcR for downregulation but does not transduce the PSY1 signal. Interestingly, endocytosis is proposed to have a prominent role in attenuating PAMP signaling [68]. Downregulation of the flagellin receptor FLS2 by its synthetic peptide ligand flg22 is a clear precedent for negative feedback in RLK signaling in plants [69]. Engagement of FLS2 and flg22 recruits the coreceptor BAK1, which concomitently promotes FLS2 signaling as well as proteosome-mediated degradation of FLS2 [70].

Importantly, the apparent negative regulation of *nPcR* by PSY1 signaling suggests a connection between PSY1 signaling and *RFO2*-mediated resistance. The mechanism that normally downregulates PSY1-activated nPcR presumably targets a common structural feature in PSY1R and nPcR, which is the extracellular n-domains of PSY1R. Conservation of the corresponding n-domains of RFO2 and PSY1R implies that RFO2 is also a target of the same negative regulation.

Because constitutive resistance of *nPcR* was not dependent on PSY1 (in *tpst nPcR*), we needed an alternative explanation for the different phenotypes of *cRFO2* and *nPcR*. Both *cRFO2* and *nPcR* encode the same c-domains of *RFO2*, so it must be the n-domains of *nPcR* that constitutively provide resistance and the n-domains of *RFO2* that constitutively provide no resistance (in the absence of FOM infection). Considering this, we hypothesize that the eLRRs of RFO2, acting as a decoy, mimic a state of the eLRRs of *PSY1R* that is already PSY1-activated and competent for downregulation. Intrinsically-activated eLRRs in RFO2 would account for their insensitivity to PSY1. Being competent for downregulation, RFO2 would be constitutively downregulated, even if no PSY1 signaling were present, and thus would normally express no resistance. On the other hand, nPcR, which needs activation by PSY1 in order to be downregulated, would constitutively express resistance in those tissues where, and at times when, there is insufficient PSY1 to fully downregulate nPcR (Figure 8B).

We present a model to explain how an effector could induce PSY1 signaling without directly engaging PSY1R, and how RFO2 could directly or indirectly perceive such an effector. By expressing an effector that inhibits the PSY1-dependent negative feedback mechanism, FOM could stabilize PSY1-activated PSY1R (Figure 8C). Just as *psy1r* and *tpst* were able to upregulated the expression of resistance by *nPcR* by abolishing PSY1-dependent downregulation, an effector could upregulate PSY1 signaling by inhibiting the downregulation of PSY1-activated PSY1R and thereby suppress host immunity. However, in plants expressing *RFO2*, the effector would likewise stabilize RFO2, acting as a decoy for the downregulation-competent PSY1R. Stabilized and upregulated RFO2 would induce robust defense response (Figure 8D).

There are two appealing aspects to this model. For one, existence of negative feedback in PSY1 signaling explains why a pathogen targets this mechanism rather than secretes a PSY1-like ligand. Chronic PSY1 signaling would be achieved more effectively by stabilizing endogenous PSY1-activated PSY1R if perception of excess PSY1-like ligand were suppressed by downregulation of the receptor. For two, RFO2 behaves as a guard protein and does not need to directly engage the effector that it recognizes. If an effector were to inhibit any component of the negative feedback mechanism, RFO2 would be activated. Thus, RFO2 functions as a guard protein for the negative feedback mechanism.

Simply changing the transcriptional context or copy number of *RFO2* in transgenic plants converted a modest quantitative resistance trait into a strong resistance gene. No aberrant visible phenotype accompanied the stronger resistance of transgenic *RFO2*, and resistance remained specific to FOM. We wonder whether gene expression rather than protein structure limits the strength of other resistance QTLs as well. Effectiveness of some qualitative gene-for-gene resistance traits is restricted, for instance, to a developmental stage, which suggests a partial deficiency in gene expression [71], [72]. Some opinion holds that resistance QTLs are in fact weak gene-for-gene resistance traits [21], [26]. If our experience of limited gene expression were commonplace, the potential utility of genes underlying resistance QTLs might be underappreciated.

Finally, our initial analysis of *RFO2* has produced a testable model to account for *RFO2*-mediated resistance. Future work should establish the biochemical nature of PSY1-dependent negative regulation of *nPcR*. In addition, identification of the relevant *F. oxysporum* PAMP(s) and/or effector(s) should prove especially useful for molecular characterization of this resistance mechanism.

## Materials and Methods

### *Arabidopsis* and plant growth conditions

Salk insertion lines and BAC DNA clones, F6I1, F20D23 and F28G4 were obtained from the *Arabidopsis* Biological Resource Center (Ohio State University, Columbus, OH). Seeds of *psy1r*, *psy1r pskr1*, *pskr1 pskr2*, *psy1r pskr1 pskr2* and *tpst* were provided by Dr. Y. Matsubayashi (Nagoya University, Nagoya, Japan). Ty-0, *rfo1* and lines 1A3 and 4D2 were derived from F_1_BC plants in [9]. Plants were grown on Jiffy7 peat pellets (Growers Solution, Cookeville, TN) under cool white fluorescent lighting with moderate intensity with 12-hr daylength and 28°C daytime and 26°C nighttime temperatures. Seedlings were grown from bleach-sterilized seeds on Petri plates with plant nutrient (PN) minimal medium and 0.8% agar alone or, for antibiotic selection, with 0.5% sucrose [73]. Transgenic seeds were selected with kanamycin (50 mg/L) or phosphinothricin (Ppt, 20 mg/L). Phytosulfokine-α was purchased from PolyPeptide Laboratories, Inc., Torrance, CA. The *Arabidopsis* Information Resource (TAIR, www.arabidopsis.org) provided reference genome sequence and annotation.

### Infections with *F. oxysporum*

FOC, FOR and FOM originate from P.H. Williams through H.C. Kistler [11], [74]. *F. oxysporum* cultures were grown on Czapek-Dox minimal medium (Oxoid Ltd., Hampshire, UK), and conidia were harvested from 3- to 5-d shaken cultures, washed 3 times and resuspended in sterile water. For infection, conidial density was adjusted to between 10^6^ to 10^8^ conidia/mL, using a hemacytometer, and 2- to 3-wk old plants were irrigated with conidial suspension or water (for mock infection). Disease symptoms were scored between 12 and 24 days post infection (dpi) using a health index (HI), previously called a disease index in [9], with ordinal ratings of one (dead) to five (unaffected) in steps of 0.5. Plants that were deemed susceptible typically had HI<3, or resistant had HI≥4 or were scored as having intermediate resistance if 3≤HI<4. For statistical analysis, plants were rank-ordered, from most susceptible to most resistance, and Mann-Whitney *U* test was used to evaluate the ranks of different genotypes. Sometimes rank-order was derived from multiple HI scores, recorded at two or more time points, in which case later HI scores had priority over earlier scores. From rank-order, plants with the lowest, highest or middle third of ranks were arbitrarily deemed susceptible, resistant or had intermediate resistance, respectively.

### Mapping *RFO2*

Linkage analysis of 80 FOM-infected progeny from the cross of 1A3 and 4D2 mapped *RFO2* between flanking SSLP markers CIW12 and F21M12 [9]. To confirm *RFO2* genotypes of plants with informative recombination breakpoints, we tested the co-segregation of Rfo2 phenotype (resistance to FOM) and genotype of an *RFO2*-linked marker in 25 to 50 progeny. If Rfo2 phenotype and marker genotype cosegregated, the genotype was *RFO2-C/T*; and, if Rfo2 phenotype and marker genotype assorted independently, the genotype was *RFO2-T/T* or *RFO2-C/C*. A fine-map position for *RFO2* was obtained by screening for recombination breakpoints in the CIW12-F21M12 interval among 200 uninfected progeny from cross (1A3×4D2) and the 248 original F_1_BC progeny (see Table S1 for description of SSLP markers for fine-mapping) [9]. In particular, Rfo2 phenotype co-segregated with *RFO2*-linked markers in progeny of 4E3 and 1B9 that have breakpoints on either side of *RFO2* (see Figure S5A). The interval between breakpoints in 4E3 and 1B9 was less than the 258 kb between SSLPs F11A6 and F17F16 (see Figure S5B).

### Cloning *RFO2*

In total, 25 Col-0 genomic restriction fragments of 3 BAC clones F6I1, F28G4 and F20D23, representing 50 of 68 genes in the *RFO2* interval (see Figure S1), were subcloned into binary vector pPZP212 [75] for *Agrobacterium tumefaciens*-mediated transformation of line 1A3. Kanamycin resistance selected for stable integration of Col-0 genomic subclones. Relative HI scores of multiple FOM-infected T_1_ and/or T_2_ transformants as well as untransformed 1A3 were used to assign Rfo2 phenotypes to subclones. A summary of all Col-0 genomic subclones and their Rfo2 phenotypes is in Figure S1.

### Sequencing *RFO2* in Ty-0

*RFO2-T* sequence (Genbank accession HQ141412) was a contig assembled from PCR-sequencing. Both strands of four overlapping PCR products amplified from Ty-0 DNA were sequenced. Primer sequences, sizes of PCR products and lengths of sequence overlap are in Table S2. Best-matched sequence to *RFO2-T* in TAIR10 reference genome database was between nucleotides 5,893,811 to 5,906,689 on chromosome 1, according to BlastN 2.2.8 search function at TAIR. DNA similarity search tool YASS (http://bioinfo.lifl.fr/yass/) assisted the hand-edited alignment of intergenic regions in Col-0 and Ty-0 sequences [76]. The percent nucleotide identity was calculated in a 75-nucleotide window centered at a nucleotide position, and mismatched nucleotides and gaps of any length in the alignment of Col-0 and Ty-0 sequences were discounted equally. Sequence of the *Arabidopsis lyrata* ortholog *AlRLP2* (gene 920636) was from the Phytozome v8.0 plant genome database.

### Genotyping

DNA markers were PCR-amplified from crude leaf preparations and analyzed as in [77]. PCR primers for genotyping *tpst*, *rfo2*, *pskr2* and *psy1r* are in Table S3.

### Genotypic and phenotypic analysis of *rfo2*

At least five plants for each of 30 homozygous Salk T-DNA lines (listed in Table S4) were infected with 5×10^7^ FOM conidia/mL. Four lines (Salk_014524, Salk_051677, Salk_049366 and Salk_097514) were crossed to Ty-0, and resulting F_1_ were infected with 10^7^ FOM conidia/mL. F_1_BC progeny of (*rfo2*×Ty-0)×Ty-0 as well as the original (Col-0×Ty-0)×Ty-0 population [9] were genotyped with *RFO1-*, *RFO2-*, *RFO3-*linked and *RFO*-unlinked Col-0-specific dominant markers (Table S5). Dominant marker primers were used with the Qiagen Multiplex PCR kit (Qiagen Inc., Valencia, CA). FOM-infected F_1_BC populations were rank-ordered using HI scores recorded at 12, 15 and 18 dpi. Lowest, middle and highest third of ranks were designated susceptible, intermediate resistance and resistant, respectively.

### Chimeric RLP and RLK transgenes

*Bam*HI and *Spe*I, or *Spe*I and *Not*I, sites were introduced to 5′ and 3′ ends of PCR-amplified sequence coding for n- or c-domains, respectively, using PCR primers with restriction sites at 5′ ends (Table S6). Sequences coding for n- and c-domains of RFO2, RLP2 and PSY1R or RLP2-T were PCR-amplified from Col-0 or Ty-0 DNA. DNA sequencing was used to verify the sequence of PCR-amplified subclones. Restriction fragments coding for n- and c-domains were ligated to *Bam*HI- and *Not*I-digested binary vector pORE-E3 to make *cRFO2*, *cRLP2*, *cRLP2-T* and *cPSY1R* expression constructs [36]. To make chimeric constructs, *Bam*HI- and *Spe*I-digested DNA for n-domains in *cRFO2*, *cRLP2*, *cRLP2-T* and *cPSY1R* expression constructs were exchanged using DNA ligation. In pORE binary vectors, gene constructs were located in T-DNA and were ready for transfer to plants using *A. tumefaciens* GV3101 [36], [78]. Resistance to Ppt selected for seedlings with stably integrated constructs, and the presence of chimeric gene sequences in transformed Ppt-resistant plants was verified by PCR.

### Visualizing and quantifying glycosidase activity in roots

Cleaning and staining of roots with 5-bromo-4-chloro-3-indoxyl-α-L-arabinofuranoside (X-Ara), 4-nitrophenyl-α-L-arabinofuranoside (NP-Ara), purchased from Gold Biotechnologies Inc. (St. Louis, MO) is described in [28]. To quantify *Fusarium*-derived arabinofuranosidase activity, freshly harvested roots were incubated with 0.04% NP-ARA for 16 h at 28°C in 30-fold excess staining solution.

### Phylogenic analysis

Coding sequences and translated sequences of *RFO2*, *RLP2*, *RLP2-T*, *PSY1R* (Atg1g72300) and *PSKR1* (At2g02220) in the TAIR10 genome and proteome databases were aligned using the Clustal method and default settings in MEGA5 [79].

## Supporting Information

Figure S1 Resistance phenotype of Col-0 subclones in the *RFO2* interval. The *RFO2* interval (258 kbp between nucleotides 5,766,000 and 6,023,500 in TAIR10 reference sequence for chromosome 1) was defined by a recombinant breakpoint in lines 4E3 between SSLPs F11A6 (at nucleotide 5,766,000) and F20D23 (at nucleotide 5,820,000), on the low end, and a recombination breakpoint in line 1B9 between SSLPs F28G4 (at nucleotide 5,943,000) and F17F16 (at nucleotide 6,023,500), on the high end. Resistance phenotypes (Rfo) of Col-0 genomic clones were tested in T_1_ and/or T_2_ transformants of line 1A3. Horizontal bars are proportional to the sequence length of subcloned Col-0 DNA and extend across their respective positions in the genomic interval below. Bars are labeled with the subclone names and, in paratheses, the sizes and gene content of subcloned sequence (to the right). Fifty of the 68 genes in the *RFO2* interval were included in at least one construct: Xba1.5 includes nucleotides 5777587 to 5789031 and genes AT1G16900, AT1G16905, AT1G16910, AT1G16916 and AT1G16920; Xba1.4 includes nucleotides 5791422 to 5801901 and genes AT1G16940, AT1G16950 and AT1G16960; Pac1.4 includes nucleotides 5810084 to 5824597 and genes AT1G17000, AT1G17010, AT1G17020 and AT1G17030; Pac1.3 includes nucleotides 5824895 to 5833233 and genes AT1G17040 and AT1G17050; Kpn1.4 includes nucleotides 5833071 to 5841463 and genes AT1G17060, AT1G17070 and AT1G17080; Xba1.3 includes nucleotides 5837955 to 5843587 and genes AT1G17070, AT1G17080 and AT1G17090; Kpn1.3 includes nucleotides 5846018 to 5854394 and genes AT1G17110 and AT1G17120; Xba1.2 includes nucleotides 5843587 to 5855420 and genes AT1G17100, AT1G17110 and AT1G17120; Pac1.2includes nucleotides 5850646 to 5866025 and genes AT1G17120, AT1G17130, AT1G17140, AT1G17145 and AT1G17147; Pac1.1 includes nucleotides 5866025 to 5878981 and genes AT1G17150, AT1G17160, AT1G17170, AT1G17180 and AT1G17190; Sal1.4 includes nucleotides 5867417 to 5882030 and genes AT1G17160, AT1G17170, AT1G17180, AT1G17190 and AT1G17200; Sal1.3 includes nucleotides 5882030 to 5891121 and genes AT1G17210 and AT1G17220; Xba1.1 includes nucleotides 5878230 to 5891727 and genes AT1G17200, AT1G17210 and AT1G17220; Kpn1.2 includes nucleotides 5886702 to 5904885 and genes AT1G17220, AT1G17232, AT1G17230, AT1G17235, AT1G17240 and AT1G17250; Sal1.2 includes nucleotides 5891121 to 5914913 and genes AT1G17232, AT1G17230, AT1G17235, AT1G17240, AT1G17250, AT1G17260, AT1G17270 and AT1G17275; Kpn1.1 includes nucleotides 5905771 to 5921349 and genes AT1G17260, AT1G17270, AT1G17275, AT1G17277, AT1G17280 and AT1G17285; Sal1.1 includes nucleotides 5933219 to 5946989 and genes AT1G17340, AT1G17345 and AT1G17350; Xma1.1 includes nucleotides 5970942 to 5986501 and genes AT1G17420, AT1G17430 and AT1G17440; Avr2.1 includes nucleotides 6007806 to 6004198 and genes no full-length gene; Nsi1.3 includes nucleotides 5898644 to 5889883 and genes AT1G17232, AT1G17230, AT1G17235 and AT1G17240; BamH1.1 includes nucleotides 5900529 to 5891012 and genes AT1G17232, AT1G17230, AT1G17235 and AT1G17240; Age1.1 includes nucleotides 5902722 to 5891905 and genes AT1G17232, AT1G17230, AT1G17235, AT1G17240 and AT1G17250; Hind3.1 includes nucleotides 5904051 to 5895229 and genes AT1G17235, AT1G17240 and AT1G17250; Nsi1.1 includes nucleotides 5907976 to 5898644 and genes AT1G17250 and AT1G17260; and, Nsi1.2 includes nucleotides 5904885 to 5898644 and gene AT1G17250.
(PDF)

Figure S2 Domain structure of RFO2. The amino acid sequence of RFO2, in single-letter code, is divided into seven alphabetically named domains. Sequence in the C domain is further subdivided into 23 leucine-rich repeats (LRRs) and a loop out sequence. The number of amino acid residues (Len) in each domain and a brief comment about each sequence are to the right of sequences. Residues corresponding to the eLRR consensus (LxxLxxLxxLxLxxNxLxGxIPxx, where x represents a nonconserved residue between conserved residues) are highlight by underlined bold type [34]. Potential N-glycosylation sites are highlighted by half-filled bold type. Conserved cysteine residues in the N- and C-cap (domains B and D, respectively) are highlighted in bold [34]. In domain E, acidic residues are highlighted in bold. In domain F, a predicted transmembrane sequence is underlined, and a conserved GxxxG motif is highlighted in bold [33].
(PDF)

Figure S3 Alignment of eLRRs (domain C) of PSY1R-like proteins and PSKR1. Alignment of the translated amino acid sequences of *PSY1R*, *RFO2*, *RLP2* (RLP2c), *RLP2-T* (RLP2t) and *PSKR1*, encoding domain C, are shown in single-letter code. All residues that are identical to PSY1R are highlighted by white type on black background. The amino acid position from the start codon is given for the leftmost residue in each line.
(PDF)

Figure S4 Alignment of PSY1R and RFO2-like RLP sequences at the carboxy-terminal ends of RLPs. Translated sequences of *RFO2*, *RLP2* (RLP2c) and *RLP2-T* (RLP2t), encoding carboxy-terminal ends of RLPs, are aligned to the translated sequence of *PSY1R* in single-letter code. Amino acid residues that are identical in >50 percent of sequences are highlighted by white type on black background. The amino acid position from the start codon is given for the leftmost residue, and asterisks are stop codons. A *Spe*I restriction site (5′-ACT-AGT-3′), which codes for the threonine (T) and serine (S) residues at the arrow, was introduced as a silent mutation into coding sequences for the creation of chimeric fusions among RLP and RLK genes.
(PDF)

Figure S5 Recombination breakpoints defining *RFO2* map position. (A) Fractions of *n* F_2_ from cross 1A3×4D2 that were susceptible (HI scores <2, open column), had intermediate resistance (2≤HI scores <4, half-filled) or were resistant (HI scores ≥4, filled) at 18 dpi. Only F_2_ of F_1_ plants 4E3, 1B9 and 5E1 from cross 1A3×4D2 that were either homozygous Ty-0 (T/T) or Col-0/Ty-0 heterzygotes (C/T) at *RFO2-*linked markers as well as C/T at *RFO1-*linked marker F19K16 are shown. (B) Genotypes, either Ty-0 (T) or Col-0 (C), at *RFO2-*linked markers (above) on the single recombinant chromosomes in F_1_ plants 4E3, 5E1 and 1B9 from cross 1A3×4D2. Marker intervals with a crossover are marked with ‘X’. Number of TAIR10 annotated open reading frames (ORFs) in marker intervals is given below. Rfo2 phenotype of F_1_ plants (on the right) was evaluated in F_2_ progeny (in A).
(PDF)

Figure S6 Root growth of *nPcR* is unaffected by PSK peptide. Two-week old seedlings of Col-0 (wild type), *psy1r*, *psy1r*, *pskr1pskr2* and *nPcR* (line 1E9) were grown from seeds sown on vertically-oriented PN agar plates with (+) or without (−) added PSK (0.1 µM). (A) Lengths of PSK-treated wild-type and psy1r roots (*n* = 20) were longer than untreated roots (*n* = 20), according to Student's *t* test (two-tailed p = 0.044 and 0.015, respectively) while length of PSK-treated and untreated *psy1r pskr1 pskr2* roots (*n* = 20) had similar lengths (*p* = 0.60). (B) Length of PSK-treated wild-type roots (*n* = 20) were longer than untreated roots (*n* = 20), according to Student's *t* test (two-tailed p = 0.005) while length of PSK-treated and untreated *nPcR* roots (*n* = 20) had similar lengths (*p* = 0.16).
(PDF)

Table S1 PCR primers for mapping and genotyping *RFO2* and *RFO1*.
(PDF)

Table S2 PCR primers and products for sequencing *RFO2* in Ty-0.
(PDF)

Table S3 PCR primers for genotyping Salk insertions.
(PDF)

Table S4 Homozygous T-DNA insertion lines in *RFO2* genetic interval.
(PDF)

Table S5 PCR primers for dominant multiplex PCR markers.
(PDF)

Table S6 PCR primers for constructing RLP and RLK genes.
(PDF)
