# Supplementary figures and images for: Arabidopsis thaliana RESISTANCE TO FUSARIUM OXYSPORUM 2 Implicates Tyrosine-Sulfated Peptide Signaling in Susceptibility and Resistance to Root Infection
Source: PLoS Genet. 2013 May 23;9(5):e1003525. doi: 10.1371/journal.pgen.1003525 (PMC3662643; doi:10.1371/journal.pgen.1003525)

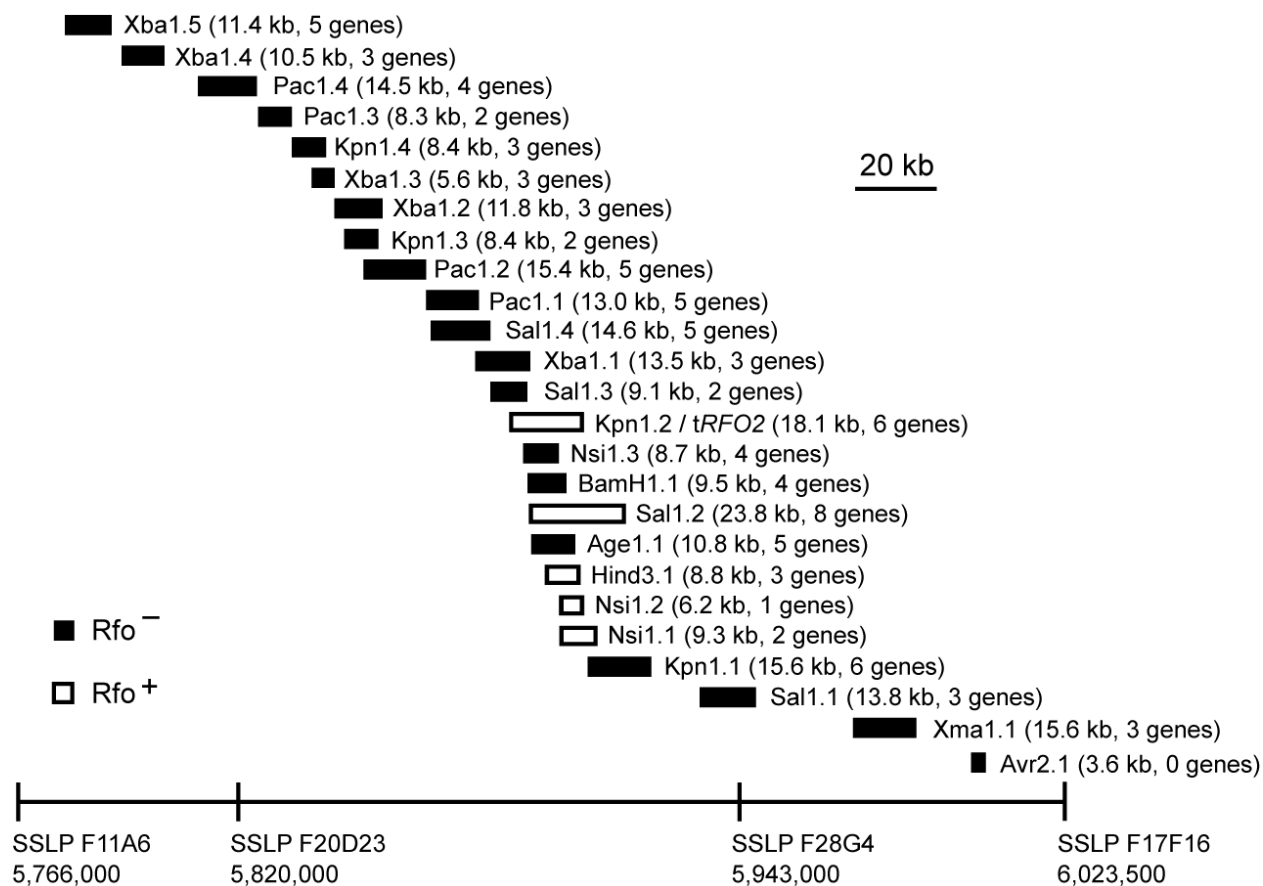

Supplement: Figure S1 — Resistance phenotype of Col-0 subclones in the RFO2 interval. The RFO2 interval (258 kbp between nucleotides 5,766,000 and 6,023,500 in TAIR10 reference sequence for chromosome 1) was defined by a recombinant breakpoint in lines 4E3 between SSLPs F11A6 (at nucleotide 5,766,000) and F20D23 (at nucleotide 5,820,000), on the low end, and a recombination breakpoint in line 1B9 between SSLPs F28G4 (at nucleotide 5,943,000) and F17F16 (at nucleotide 6,023,500), on the high end. Resistance phenotypes (Rfo) of Col-0 genomic clones were tested in T1 and/or T2 transformants of line 1A3. Horizontal bars are proportional to the sequence length of subcloned Col-0 DNA and extend across their respective positions in the genomic interval below. Bars are labeled with the subclone names and, in paratheses, the sizes and gene content of subcloned sequence (to the right). Fifty of the 68 genes in the RFO2 interval were included in at least one construct: Xba1.5 includes nucleotides 5777587 to 5789031 and genes AT1G16900, AT1G16905, AT1G16910, AT1G16916 and AT1G16920; Xba1.4 includes nucleotides 5791422 to 5801901 and genes AT1G16940, AT1G16950 and AT1G16960; Pac1.4 includes nucleotides 5810084 to 5824597 and genes AT1G17000, AT1G17010, AT1G17020 and AT1G17030; Pac1.3 includes nucleotides 5824895 to 5833233 and genes AT1G17040 and AT1G17050; Kpn1.4 includes nucleotides 5833071 to 5841463 and genes AT1G17060, AT1G17070 and AT1G17080; Xba1.3 includes nucleotides 5837955 to 5843587 and genes AT1G17070, AT1G17080 and AT1G17090; Kpn1.3 includes nucleotides 5846018 to 5854394 and genes AT1G17110 and AT1G17120; Xba1.2 includes nucleotides 5843587 to 5855420 and genes AT1G17100, AT1G17110 and AT1G17120; Pac1.2includes nucleotides 5850646 to 5866025 and genes AT1G17120, AT1G17130, AT1G17140, AT1G17145 and AT1G17147; Pac1.1 includes nucleotides 5866025 to 5878981 and genes AT1G17150, AT1G17160, AT1G17170, AT1G17180 and AT1G17190; Sal1.4 includes nucleotides 5867417 to 5882030 and genes AT1G17160, [file pgen.1003525.s001.pdf]

A

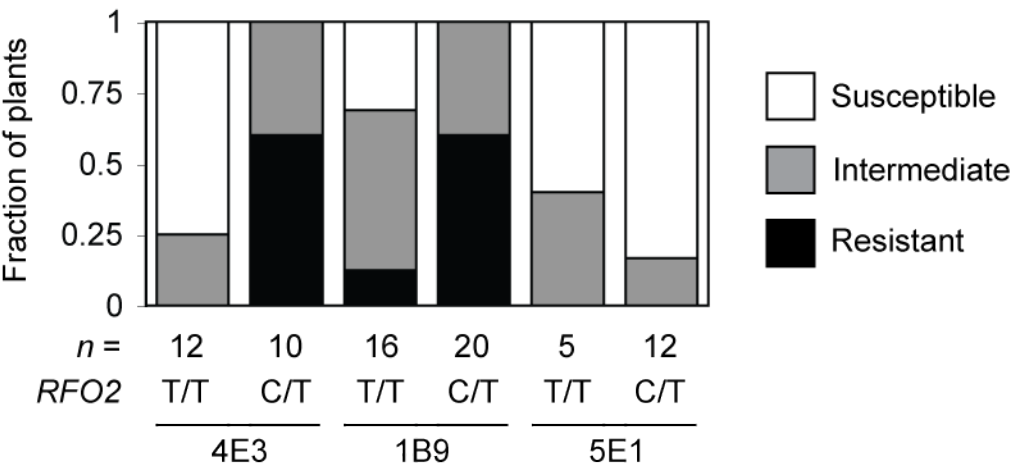

B

|      | F2H15 | F11A6 | F28G4 | F20D23 | F17F16 | Rfo2 |
|------|-------|-------|-------|--------|--------|------|
| 4E3  | T     | T     | X     | C      | C      | +    |
| 5E1  | C     | X     | T     | T      | T      | -    |
| 1B9  | C     | C     | C     | C      | X      | +    |
| ORFs | 39    | 19    | 36    | 13     |        |      |

Supplement: Figure S5 — Recombination breakpoints defining RFO2 map position. (A) Fractions of n F2 from cross 1A3×4D2 that were susceptible (HI scores <2, open column), had intermediate resistance (2≤HI scores <4, half-filled) or were resistant (HI scores ≥4, filled) at 18 dpi. Only F2 of F1 plants 4E3, 1B9 and 5E1 from cross 1A3×4D2 that were either homozygous Ty-0 (T/T) or Col-0/Ty-0 heterzygotes (C/T) at RFO2-linked markers as well as C/T at RFO1-linked marker F19K16 are shown. (B) Genotypes, either Ty-0 (T) or Col-0 (C), at RFO2-linked markers (above) on the single recombinant chromosomes in F1 plants 4E3, 5E1 and 1B9 from cross 1A3×4D2. Marker intervals with a crossover are marked with ‘X’. Number of TAIR10 annotated open reading frames (ORFs) in marker intervals is given below. Rfo2 phenotype of F1 plants (on the right) was evaluated in F2 progeny (in A). (PDF) [file pgen.1003525.s005.pdf]

**A**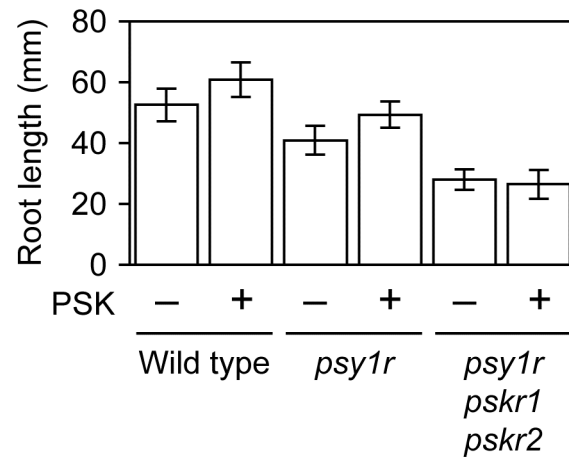**B**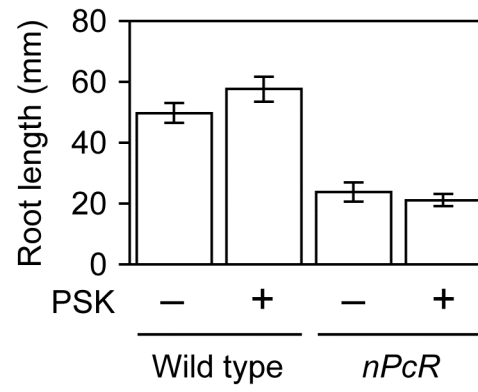

Supplement: Figure S6 — Root growth of nPcR is unaffected by PSK peptide. Two-week old seedlings of Col-0 (wild type), psy1r, psy1r, pskr1pskr2 and nPcR (line 1E9) were grown from seeds sown on vertically-oriented PN agar plates with (+) or without (−) added PSK (0.1 µM). (A) Lengths of PSK-treated wild-type and psy1r roots (n = 20) were longer than untreated roots (n = 20), according to Student's t test (two-tailed p = 0.044 and 0.015, respectively) while length of PSK-treated and untreated psy1r pskr1 pskr2 roots (n = 20) had similar lengths (p = 0.60). (B) Length of PSK-treated wild-type roots (n = 20) were longer than untreated roots (n = 20), according to Student's t test (two-tailed p = 0.005) while length of PSK-treated and untreated nPcR roots (n = 20) had similar lengths (p = 0.16). (PDF) [file pgen.1003525.s006.pdf]
