# Supplementary material for: Arabidopsis thaliana RESISTANCE TO FUSARIUM OXYSPORUM 2 Implicates Tyrosine-Sulfated Peptide Signaling in Susceptibility and Resistance to Root Infection
Source: PLoS Genet. 2013 May 23;9(5):e1003525. doi: 10.1371/journal.pgen.1003525 (PMC3662643; doi:10.1371/journal.pgen.1003525)
Supplement: Figure S2 — Domain structure of RFO2. The amino acid sequence of RFO2, in single-letter code, is divided into seven alphabetically named domains. Sequence in the C domain is further subdivided into 23 leucine-rich repeats (LRRs) and a loop out sequence. The number of amino acid residues (Len) in each domain and a brief comment about each sequence are to the right of sequences. Residues corresponding to the eLRR consensus (LxxLxxLxxLxLxxNxLxGxIPxx, where x represents a nonconserved residue between conserved residues) are highlight by underlined bold type [34]. Potential N-glycosylation sites are highlighted by half-filled bold type. Conserved cysteine residues in the N- and C-cap (domains B and D, respectively) are highlighted in bold [34]. In domain E, acidic residues are highlighted in bold. In domain F, a predicted transmembrane sequence is underlined, and a conserved GxxxG motif is highlighted in bold [33]. (PDF) [file pgen.1003525.s002.pdf]

Domain	Amino acid sequence	Len	Comment
A	MIGILLQCVLFISVLSIAVSEA	22	Signal peptide
B	LCNSQDRESLLWFSGNVSSSVSPLNWNPSIDCCSWEGI	40	N-cap
C	DDSPDSHITAISLPFRALYG KLP	23	LRR1
	LSVLRLHHLSQLNLSHNRLSG HLP	24	LRR2
	SGFLSALDQLKVLDLSYNSLDG ELP	25	LRR3
	VEQTFRNGSNRCFPIRIVDLSSNFLQGEILP	31	LRR4
	SSIFMQGTFDLISFNVSKNSFTG SIP	26	LRR5
	SFMCKSSPQLSKLDFSYNDFTG NIP	25	LRR6
	QGLGRCLKLSVLQAGFNNISG EIP	24	LRR7
	SDIYNLSELEQLFLPVNHLSG KIN	24	LRR8
	DDITHLTKLKSLELYSNHLGG EIP	24	LRR9
	MDIGQLSRLQSLQLHINNITG TVP	24	LRR10
	PSLANCTNLVKLNLRLNRLEG TLS	24	LRR11
	ELDFSRFQSLSILDLGNNSFSG DFP	25	LRR12
	WRVHSCKSLSAMRFASNKLTGQISP	25	LRR13
	HVLELESLSILSLSDNKLM NIT	22	LRR14
	GALGILQGCRNLSTLLIGKNFYNE TFP	27	LRR15
	SDKDLISSDGFPNLQIFASGGSGLRG EIP	29	LRR16
	AWLIKLKSLAVIDLSHNQLVG SIP	24	LRR17
	GWLGTFPHLFYIDLSENLLSG ELP	24	LRR18
	KDLFQLKALM	10	LRR19
	SQKAYDATERNYLKLPVFVSPNNVTTHQQYNQLFSLPP	38	loop out
	GIYIRRNNLKG SIP	14	LRR19
	IEVGQLKVLHVLELSHNYLSG IIP	24	LRR20
	HELSKLTSLERLDLSNNHLSG RIP	24	LRR21
	WSLTSLHYMSYFNVVNNSLDG PIP	24	LRR22
	TGSQFDTFPQANFKGNPLLCGGIL	24	LRR23
D	LTSCKASTKLPATTTNKA	18	C-cap
E	DTEDEEELK	9	Acidic
F	FIFILGVATGFFVSYCFYWCFF	22	Transmembrane
G	ARLDAFISK	9	Cytoplasmic tail
