# Supplementary material for: Arabidopsis thaliana RESISTANCE TO FUSARIUM OXYSPORUM 2 Implicates Tyrosine-Sulfated Peptide Signaling in Susceptibility and Resistance to Root Infection
Source: PLoS Genet. 2013 May 23;9(5):e1003525. doi: 10.1371/journal.pgen.1003525 (PMC3662643; doi:10.1371/journal.pgen.1003525)
Supplement: Figure S3 — Alignment of eLRRs (domain C) of PSY1R-like proteins and PSKR1. Alignment of the translated amino acid sequences of PSY1R, RFO2, RLP2 (RLP2c), RLP2-T (RLP2t) and PSKR1, encoding domain C, are shown in single-letter code. All residues that are identical to PSY1R are highlighted by white type on black background. The amino acid position from the start codon is given for the leftmost residue in each line. (PDF) [file pgen.1003525.s003.pdf]

```
PSY1R   94  VTSIILSSRGLSGNLPSSVLDLQRLSRLDLSHNRLSGPLPPGFLSALDQLLVLDLSYNSFKGELPLQQSFGNGSNGIFPIQTVDLSSNLLEGEILSSSVF
RFO2    98  ITAISLPFRALYGKLPLSVLRLHHLSQLNLSHNRLSGHLPSGFLSALDQLKVLDLSYNSLDGELPVEQTFRNGSNRCFPIRIVDLSSNFLQGEILPSSIF
RLP2c   92  VTVISLPSRGLSGTLASSVQNIHRLSRLDLSYNRLSGPLPPGFFSTLDQLMILNLSYNSFNGELPLEQAFGNESNRFFSIQTLDLSSNLLEGEILRSSVY
RLP2t   70  VTTISLPSRGLSGTLTSSLQNLHRLSRLDLSYNHLSGPLPQVLFSTLNQLMVLNLSYNSFNGELPLEQAFGNESNRFFVIQTLDLSSNLLQGQILRSSVY
PSKR1   78  VIRLELGNKKLSGKLSESLGKLDEIRVLNLSRNFIKDSIPLSIFN-LKNLQTLDLSSNDLSGGIPTSINLP-----------------------------

PSY1R  194  LQGAFNLTSFNVSNNSFTGSIPSFMCTASPQLTKLDFSYNDFSGDLSQELSRCSRLSVLRAGFNNLSGEIPKEIYNLPELEQLFLPVNRLSGKIDNGITR
RFO2   198  MQGTFDLISFNVSKNSFTGSIPSFMCKSSPQLSKLDFSYNDFTGNIPQGLGRCLKLSVLQAGFNNISGEIPSDIYNLSELEQLFLPVNHLSGKINDDITH
RLP2c  192  LQGTINLISFNVSNNSFTGPIPSFMCRSSPQLSKLDFSYNDFSGHISQELGRCLRLTVLQAGFNNLSGVIPSEIYNLSELEQLFLPANQLTGKIDNNITR
RLP2t  170  LLGAINLISFNVSNNSFTGPIPSFMCRSSPQLSKLDFSYNDFSDHISQELSRCLGLRVLRAGFNSLSGEIPSEIYNLSELEQLFLPVNRLSGKIDDDITR
PSKR1  148  -----ALQSFDLSSNKFNGSLPSHICHNSTQIRVVKLAVNYFAGNFTSGFGKCVLLEHLCLGMNDLTGNIPEDLFHLKRLNLLGIQENRLSGSLSREIRN

PSY1R  294  LTKLTLLELYSNHIEGEIPKDIGKLSKLSSLQLHVNNLMGSIPVSLANCTKLVKLNLRVNQLGGTLSAIDFSRFQSLSILDLGNNSFTGEFPSTVYSCKM
RFO2   298  LTKLKSLELYSNHLGGEIPMDIGQLSRLQSLQLHINNITGTVPPSLANCTNLVKLNLRLNRLEGTLSELDFSRFQSLSILDLGNNSFSGDFPWRVHSCKS
RLP2c  292  LRKLTSLALYSNHLEGEIPMDIGNLSSLRSLQLHINNINGTVPLSLANCTKLVKLNLRVNQLGGGLTELEFSQLQSLKVLDLGNNSFTGALPDKIFSCKS
RLP2t  270  LSKLTTLDLYFNHLEGDIPVGIGKLSSLRSLQLHINNLTGSIPLSLANCTNLVKLNLRVNHLGGSLTELDFSQFQSLRILDLGNNSFTGDFPDKVYSCKS
PSKR1  243  LSSLVRLDVSWNLFSGEIPDVFDELPQLKFFLGQTNGFIGGIPKSLANSPSLNLLNLRNNSLSG-RLMLNCTAMIALNSLDLGTNRFNGRLPENLPDCKR

PSY1R  394  MTAMRFAGNKLTGQISPQVLELESLSFFTFSDNKMTNLTGALSILQGCKKLSTLIMAKNFYDETVPSNKDFLRSDGFPSLQIFGIGACRLTGEIPAWLIK
RFO2   398  LSAMRFASNKLTGQISPHVLELESLSILSLSDNKLMNITGALGILQGCRNLSTLLIGKNFYNETFPSDKDLISSDGFPNLQIFASGGSGLRGEIPAWLIK
RLP2c  392  LTAIRFAGNKLTGEISPQVLELESLSFMGLSDNKLTNITGALSILQGCRKLSTLILAKNFYDETVPSKEDFLSPDGFPKLRIFGVGACRLRGEIPAWLIN
RLP2t  370  LTAIRFAGNKLTGQISPQVLELESLSFMSFSDNKLTNITGALSILQGCRKLSTLIIANNFYDETVPINEDFVAPDGFPKLQIFGIGGSRLKGEIPAWLIK
PSKR1  342  LKNVNLARNTFHGQVPESFKNFESLSYFSLSNSSLANISSALGILQHCKNLTTLVLTLNFHGEALPDD----SSLHFEKLKVLVVANCRLTGSMPRWLSS

PSY1R  494  LQRVEVMDLSMNRFVGTIPGWLGTLPDLFYLDLSDNFLTGELPKELFQLRALMSQKAYDATERNYLELPVFVNPNNVTTNQQYNQLSSLPPTIYIKRNNL
RFO2   498  LKSLAVIDLSHNQLVGSIPGWLGTFPHLFYIDLSENLLSGELPKDLFQLKALMSQKAYDATERNYLKLPVFVSPNNVTTHQQYNQLFSLPPGIYIRRNNL
RLP2c  492  LNKVEVMDLSMNRFVGSIPGWLGTLPDLFYLDLSDNLLTGELPKELFQLRALMSQK---ITENNYLELPIFLNPNNVTTNQQYNKLYSFPPTIYIRRNNL
RLP2t  470  IKSLEVLDLSLNRFEGLIPGWLGTLPNLFYMDLSDNLLSGELPKEIFHLRALMSQQAYDATERNYLELPVFVNPNNVTTNQQYNQLSSLPPSIHIRRNKL
PSKR1  438  SNELQLLDLSWNRLTGAIPSWIGDFKALFYLDLSNNSFTGEIPKSLTKLESLTSRN--ISVNEPSPDFPFFMKRNESARALQYNQIFGFPPTIELGHNNL

PSY1R  594  TGTIPVEVGQLKVLHILELLGNNFSGSIPDELSNLTNLERLDLSNNNLSGRIPWSLTGLHFLSYFNVANNTLSGPIPTGTQFDTFPKANFEGNPLLCGGV
RFO2   598  KGSIPIEVGQLKVLHVLELSHNYLSGIIPHELSKLTSLERLDLSNNHLSGRIPWSLTSLHYMSYFNVVNNSLDGPIPTGSQFDTFPQANFKGNPLLCGGI
RLP2c  589  TGSIPVEVGQLKVLHILELLGNNLSGSIPDELSNLTNLERLDLSNNNLSGSIPWSLTNLNFLSYFNVANNSLEGPIPSEGQFDTFPKANFEGNPLLCGGV
RLP2t  570  TGSIPVEVGQLKVIHVLELLGNNLSGSIPEELSNLTNLERLDLSNNNLSGRIPWSLTSLHFMSYFNVANNTLEGTIPRGSLFDTFPKAYFEGNPLLCGGV
PSKR1  536  SGPIWEEFGNLKKLHVFDLKWNALSGSIPSSLSGMTSLEALDLSNNRLSGSIPVSLQQLSFLSKFSVAYNNLSGVIPSGGQFQTFPNSSFESN-HLCGEH
```
