# Supplementary material for: Arabidopsis thaliana RESISTANCE TO FUSARIUM OXYSPORUM 2 Implicates Tyrosine-Sulfated Peptide Signaling in Susceptibility and Resistance to Root Infection
Source: PLoS Genet. 2013 May 23;9(5):e1003525. doi: 10.1371/journal.pgen.1003525 (PMC3662643; doi:10.1371/journal.pgen.1003525)
Supplement: Figure S4 — Alignment of PSY1R and RFO2-like RLP sequences at the carboxy-terminal ends of RLPs. Translated sequences of RFO2, RLP2 (RLP2c) and RLP2-T (RLP2t), encoding carboxy-terminal ends of RLPs, are aligned to the translated sequence of PSY1R in single-letter code. Amino acid residues that are identical in >50 percent of sequences are highlighted by white type on black background. The amino acid position from the start codon is given for the leftmost residue, and asterisks are stop codons. A SpeI restriction site (5′-ACT-AGT-3′), which codes for the threonine (T) and serine (S) residues at the arrow, was introduced as a silent mutation into coding sequences for the creation of chimeric fusions among RLP and RLK genes. (PDF) [file pgen.1003525.s004.pdf]

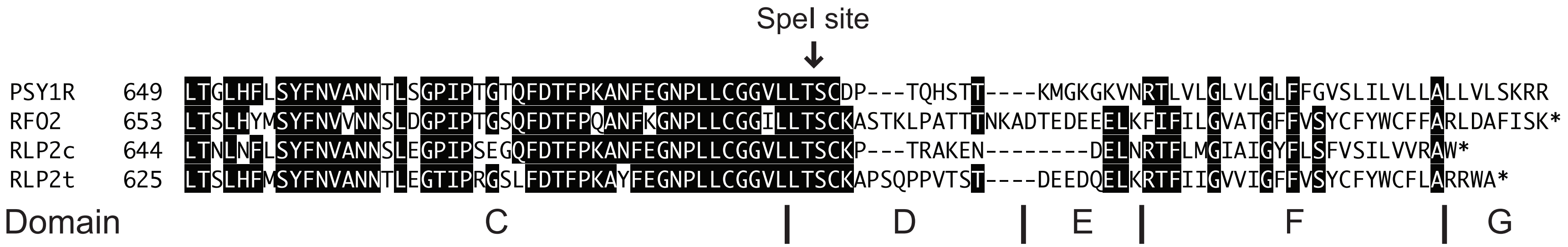
SpeI site
PSY1R 649 LTGLHFLSYFNVANNTLSGPIPTGTQFDTFPKANFEGNPLLCGGVLLTSCDP---TQHSTT----KMGKGKVNRTLVLGLVLGLFFGVSLILVLLALLVLSKRR
RFO2 653 LTSLHYMSYFNVVNNSLDGPIPTGSQFDTFPQANFKGNPLLCGGILLTSCKASTKLPATTTNKADTEDEEELKFIFILGVATGFFVSYCFYWCFFARLDAFISK*
RLP2c 644 LTNLNFLSYFNVANNSLEGPIPSEGQFDTFPKANFEGNPLLCGGVLLTSCKP---TRAKEN---------DELNRTFLMGIAIGYFLSFVSILVVRAW*
RLP2t 625 LTSLHFMSYFNVANNTLEGTIPRGSLFDTFPKAYFEGNPLLCGGVLLTSCKAPSQPPVTST----DEEDQELKRTFIIGVVIGFFVSYCFYWCFLARRWA*
Domain
C
D
E
F
G
