# Supplementary material for: Arabidopsis thaliana RESISTANCE TO FUSARIUM OXYSPORUM 2 Implicates Tyrosine-Sulfated Peptide Signaling in Susceptibility and Resistance to Root Infection
Source: PLoS Genet. 2013 May 23;9(5):e1003525. doi: 10.1371/journal.pgen.1003525 (PMC3662643; doi:10.1371/journal.pgen.1003525)
Supplement: Table S1 — PCR primers for mapping and genotyping RFO2 and RFO1. (PDF) [file pgen.1003525.s007.pdf]

Table S1. PCR primers for mapping and genotyping *RFO2* and *RFO1*

Marker	Primer	Oligonucleotide sequence	Chr.[a]	Nucleotide[b]
F11A6	F11A6.1-F	5'-gattgaagcaacttgatcgatg-3'	1	6,098,239
	F11A6.1-R	5'-tagagagagaaaaaggccaagg-3'	1	6,098,392
F20D23	F20D23-F	5'-ttttttaccaaccccttatatatc-3'	1	5,819,738
	F20D23-F	5'-aacggaaaaaaaaattgtacaatg-3'	1	5,819,580
F28G4	F28G4.2-F	5'-ttgagcgcattccacagaaac-3'	1	5,943,414
	F28G4.2-R	5'-caggtttaagcatctgctccg-3'	1	5,942,863
F17F16	F17F16-F	5'-gcgtctgctttaggtagccaca-3'	1	5,734,256
	F17F16-R	5'-agtaaggaaatttctccatttc-3'	1	5,734,144
T8K14	T8K14-F	5'-agtcgagggagagttaaaacttag-3'	1	29,910,068
	T8K14-R	5'-ttgtatagttcgaaaaagtaagcg-3'	1	29,909,933
RFO2CnT	RFO2CnT-1	5'-ttgaaggaaaccccttgttg-3'	1	5,896,685
	RFO2CnT-2	5'-ccaggcacgcaccactaaga-3'	1	5,896,531
	RFO2CnT-3	5'-cttaagcccatctcctagcaagaaag-3'	1	—[c]

[a] Abbreviation for chromosome number
[b] Position of most 5' nucleotide in the Arabidopsis TAIR10 genome sequence annotation
[c] Oligonucleotide sequence is specific for the *RFO2* region in Ty-0.
