# Supplementary material for: Arabidopsis thaliana RESISTANCE TO FUSARIUM OXYSPORUM 2 Implicates Tyrosine-Sulfated Peptide Signaling in Susceptibility and Resistance to Root Infection
Source: PLoS Genet. 2013 May 23;9(5):e1003525. doi: 10.1371/journal.pgen.1003525 (PMC3662643; doi:10.1371/journal.pgen.1003525)
Supplement: Table S2 — PCR primers and products for sequencing RFO2 in Ty-0. (PDF) [file pgen.1003525.s008.pdf]

Table S2. PCR Primers and products for sequencing *RFO2* in Ty-0

#[a]	Primer	Oligonucleotide sequence	Nucl.[b]	Length[c]	Overlap[d]
1	230RP	5’-tcgtggatgcaaccagaaact-3’	< 0[e]	> 1,900	
	235stopR	5’-aaaactgcagagctcgagcgtaatcgtcgtgatctgtgta-3’	1,900		
					508
2	230CF1	5’-tcgttactcttgataagctcgttga-3’	1,392	2,648	
	250MF	5’-tttcggagctcgagcaactct-3’	4,040		
					360
3	240MR2	5’-cttgttgcctgcaaatctgat-3’	3,680	3,144	
	250NF1	5’-catctgtactgtccaccaagcg-3’	6,824		
					480
4	250NR3	5’-ctgctgcagctcttatggctc-3’	6,344	> 1,967	
	260R1	5’-ggccaagcctaccaagctaaa-3’	> 8,311		

[a] Designation of PCR-amplified DNA product
[b] Nucleotide in Ty-0 DNA, Genbank accession HQ141412, at 3’ end of primer
[c] Length of PCR-amplified DNA product from Ty-0 in basepairs
[d] Length of DNA sequence overlap between DNA fragments (above and below)
[e] Primer anneals to sequence that is adjacent to sequence in Genbank accession HQ141412.
