# Supplementary material for: Arabidopsis thaliana RESISTANCE TO FUSARIUM OXYSPORUM 2 Implicates Tyrosine-Sulfated Peptide Signaling in Susceptibility and Resistance to Root Infection
Source: PLoS Genet. 2013 May 23;9(5):e1003525. doi: 10.1371/journal.pgen.1003525 (PMC3662643; doi:10.1371/journal.pgen.1003525)
Supplement: Table S3 — PCR primers for genotyping Salk insertions. (PDF) [file pgen.1003525.s009.pdf]

Table S3. PCR primers for genotyping Salk insertions

Name[a]	Mutant	TAIR10 gene	Suffix[b]	Primer sequences
Salk_009847	*tpst*	At1g08030	LP	5'-gcaagttgttagccacacatg-3'
			RP	5'-aggcgcatcaagatatttgtg-3'
Salk_024464	*pskr2*	At5g53890	LP	5'-ctaagcgaattgctcgaaatg-3'
			RP	5'-aatttgcttccacaatgttgc-3'
Salk_051677	*rfo2*	At1g17250	LP	5'-ggattttgataaagcctgcaca-3'
			RP	5'-tagatttgtcccagaagcagc-3'
Salk_072802	*psy1r*	At1g72300	LP	5'-tttgttgcttggtaccgtttc-3'
			RP	5'-tctggcaaaattgataatggg-3'
Salk_077975	*rfo1*	At1g79670	LP	5'-gagatttaatgtgaacaagtcc-3'
			RP	5'-cgttggtgaatagtcaatttcc-3'

[a] Primer name is given for the detected T-DNA insertion allele. Primer pairs yield wild-type PCR products. Addition of LBb1, 5'-gcgtggaccgcttgctgcaact-3', to PCR reactions also yields insertion-specific products as described elsewhere (http://signal.salk.edu/tdnaprimers.2.html).
[b] Suffixes of primer names in pairs are either '-LP' or '-RP'. RBb1 (5'-acgttgcggttctgtcagttcc-3') was used to amplify sequence flanking the T-DNA right border.
