# Supplementary material for: Arabidopsis thaliana RESISTANCE TO FUSARIUM OXYSPORUM 2 Implicates Tyrosine-Sulfated Peptide Signaling in Susceptibility and Resistance to Root Infection
Source: PLoS Genet. 2013 May 23;9(5):e1003525. doi: 10.1371/journal.pgen.1003525 (PMC3662643; doi:10.1371/journal.pgen.1003525)
Supplement: Table S4 — Homozygous T-DNA insertion lines in RFO2 genetic interval. (PDF) [file pgen.1003525.s010.pdf]

Table S4. Homozygous T-DNA insertion lines in *RFO2* genetic interval

ABRC order number	TAIR10 gene
SALK_001977C	At1g16890
SALK_009736C	At1g17130
SALK_009815C	At1g16980
SALK_010091C	At1g16930
SALK_014524C	At1g17200
SALK_019569C	At1g17260
SALK_025224C	At1g17480
SALK_037965C	At1g17495
SALK_042510C	At1g17430
**SALK_049366C**[a]	**At1g17240**
**SALK_051677C**	**At1g17250**
SALK_053775C	At1g17380
SALK_066856C	At1g17110
SALK_072749C	At1g17410
SALK_075228C	At1g17500
SALK_080117C	At1g16900
SALK_091444C	At1g17030
**SALK_097514C**	**At1g17230**
SALK_104566C	At1g17330
SALK_104663C	At1g17080
SALK_107662C	At1g17290
SALK_112785C	At1g17345
SALK_120416C	At1g17060
SALK_123114C	At1g16970
SALK_126802C	At1g16940
SALK_127663C	At1g17100
SALK_130749C	At1g17150
SALK_132293C	At1g17440
SALK_137479C	At1g17160
SALK_147830C	At1g17420

[a] Salk lines in bold have insertions in the *RFO2* region as defined by common sequence in Kpn1.2 and Sal1.2 genomic subclones.
