# Supplementary material for: Arabidopsis thaliana RESISTANCE TO FUSARIUM OXYSPORUM 2 Implicates Tyrosine-Sulfated Peptide Signaling in Susceptibility and Resistance to Root Infection
Source: PLoS Genet. 2013 May 23;9(5):e1003525. doi: 10.1371/journal.pgen.1003525 (PMC3662643; doi:10.1371/journal.pgen.1003525)
Supplement: Table S5 — PCR primers for dominant multiplex PCR markers. (PDF) [file pgen.1003525.s011.pdf]

Table S5. PCR primers for dominant multiplex PCR markers

Marker	Primer	Gene[a]	Oligonucleotide sequence	Chr.[b]	Nucleotide[c]
CHR1.3	CHR1.3-F	*RFO2*	5'-ttaaatcaggctggccctaatgccttt-3'	1	7,969,890
	CHR1.3-R		5'-gtggattttgcagtgctagttccctca-3'	1	7,970,182
CHR1.9	CHR1.9-F	*RFO1*	5'-gaaaaggagatgccgtttcgtccctac-3'	1	30,425,560
	CHR1.9-R		5'-caaagtcggtttcgatttgggttttgt-3'	1	30,426,060
CHR3.3	CHR3.3-F	*RFO3*	5'-tgatggagtgaaaaagcatgtccattg-3'	3	5,440,460
	CHR3.3-R		5'-ctgaaagtataaccaaagcgcgagcaa-3'	3	5,440,672
CHR3.8	CHR3.8-F	none	5'-gtattggcgactccaaatcccattcac-3'	3	22558313
	CHR3.8-R		5'-gtcctgtgatagcctcacccgcttct-3'	3	22558677

[a] *RFO* QTL is linked to marker CHR*x.x*.
[b] Abbreviation for chromosome number
[c] Position of most 5' nucleotide in the Arabidopsis TAIR10 reference genome. Size of PCR product is the difference between nucletide positions of the primer pair.
