# Supplementary material for: Arabidopsis thaliana RESISTANCE TO FUSARIUM OXYSPORUM 2 Implicates Tyrosine-Sulfated Peptide Signaling in Susceptibility and Resistance to Root Infection
Source: PLoS Genet. 2013 May 23;9(5):e1003525. doi: 10.1371/journal.pgen.1003525 (PMC3662643; doi:10.1371/journal.pgen.1003525)
Supplement: Table S6 — PCR primers for constructing RLP and RLK genes. (PDF) [file pgen.1003525.s012.pdf]

Table S6. PCR primers for constructing RLP and RLK genes

End[a]	Gene	Primer Name	Primer Sequence[b]
N	*cRLP2-T*	Ty0240startBamH1	5'-cgc**ggatcc**atgcttttcttcctccttctc-3'
		240bTMRSpe1	5'-ctagcgg**actagt**cagcagtacaccaccgcacaa-3'
N	*cRLP2*	240startFBamH1	5'-cgc**ggatcc**atgagatccaaagccaaaggtc-3'
		240bTMRSPE1	5'-ctagcgg**actagt**cagcagtacaccaccgcacaa-3'
N	*cRFO2*	250startFBamH1	5'-cgc**ggatcc**atgactaatgaggggagattc-3'
		250bTMRSPE1	5'-ctagcgg**actagt**cagcagtatgccaccgcacaa-3'
N	*cPSY1R*	72300startFBamH1	5'-cgc**ggatcc**atgattgacgagaagatgag-3'
		72300bTMRSpe1	5'-ctagcgg**actagt**tagcagtacaccaccgcacagc-3'
C	*cRLP2-T*	Ty0240bTMFSpe1	5'-ctagcgg**actagt**tgcaaggctccaagtcagccgcc-3'
		Ty0240stopRNot1	5'-aaggaaaaaa**gcggccgc**ttaagcccatctcctagcaagaaag-3'
C	*cRLP2*	240bTMFSpe1	5'-ctagcgg**actagt**tgtaaacctactagagccaaag-3'
		240stopRNot1	5'-aaggaaaaaa**gcggccgc**tcaccaggcacgcaccactaagatc-3'
C	*cRFO2*	250bTMFSpe1	5'-ctagcgg**actagt**tgcaaggcttcaacaaagctgc-3'
		250stopRNot1	5'-aaggaaaaaa**gcggccgc**ttacttactaatgaaagcatcgag-3'
C	*cPSY1R*	72300bTMFSpe1	5'-ctagcgg**actagt**tgcgatccaactcagcactctac-3'
		72300stopRNot1	5'-aaggaaaaaa**gcggccgc**ttacgtctcttcttcttcttcagg-3'

[a] PCR primer pairs direct amplification of amino-terminal (N) and carboxyl-terminal (C) coding sequences of the target gene.

[b] Restriction sites, *Bam*HI, *Spe*I and *Not*I, included in primer sequence for subcloning coding sequence of N- and C-terminal domains are highlighted in bold.
